# Lupin (*Lupinus* spp.)-Fortified Bread: A Sustainable, Nutritionally, Functionally, and Technologically Valuable Solution for Bakery

**DOI:** 10.3390/foods11142067

**Published:** 2022-07-12

**Authors:** Loredana Plustea, Monica Negrea, Ileana Cocan, Isidora Radulov, Camelia Tulcan, Adina Berbecea, Iuliana Popescu, Diana Obistioiu, Ionela Hotea, Gabriel Suster, Adriana Elena Boeriu, Ersilia Alexa

**Affiliations:** 1 Faculty of Food Engineering, Banat’s University of Agricultural Sciences and Veterinary Medicine, Calea Aradului No. 119, 300645 Timisoara, Romania; loredanapaven@usab-tm.ro (L.P.); ileanacocan@usab-tm.ro (I.C.); ersiliaalexa@usab-tm.ro (E.A.); 2 Faculty of Agriculture, Banat’s University of Agricultural Sciences and Veterinary Medicine, Calea Aradului No. 119, 300645 Timisoara, Romania; isidora_radulov@usab-tm.ro (I.R.); adina_berbecea@usab-tm.ro (A.B.); iuliana_popescu@usab-tm.ro (I.P.); 3 Faculty of Horticulture and Forestry, Banat’s University of Agricultural Sciences and Veterinary Medicine, Calea Aradului No. 119, 300641 Timisoara, Romania; cameliatulcan@usab-tm.ro; 4 Faculty of Veterinary Medicine, Banat’s University of Agricultural Sciences and Veterinary Medicine, Calea Aradului No. 119, 300641 Timisoara, Romania; dianaobistioiu@usab-tm.ro (D.O.); ionelahotea@usab-tm.ro (I.H.); 5 Faculty of Management and Ago-Tourism, Banat’s University of Agricultural Sciences and Veterinary Medicine, Calea Aradului No. 119, 300645 Timisoara, Romania; gabrielsuster@usab-tm.ro; 6 Faculty of Food and Tourism, Transilvania University of Brasov, 148 Castelului Str., 500014 Brasov, Romania; adriana.boeriu@unitbv.ro

**Keywords:** lupin flour, wheat–lupin bread, sensory score, antioxidant activity, fatty acids, flavonoids, Mixolab

## Abstract

The purpose of this paper is to evaluate the nutritional, phytochemical, rheological, technological, and sensory properties of wheat flour dough and bread under a replacement of lupin flour at level 10, 20, and 30%. In this sense, the proximate composition, fatty acids profile, the content in total polyphenols content (TPC), antioxidant activity (AA), and flavonoids content (TFC) of lupin; wheat and flour composites; and the bread obtained from them were determined. The rheological properties of the dough using the Mixolab system were also evaluated. The results showed an improvement in the nutritional properties of bread with addition of lupin in the composite flour, especially in terms of proteins, lipids, and mineral substances and a significant increases of functional attributes, such as TPC, TFC, and AA, which recorded the highest values in the bread with 30% lupin flour (76.50 mg GAE/100 g, 8.54 mg QE/100 g, 54.98%). The decrease of lupin bread volume compared to wheat bread ranged between 0.69–7.37%, porosity between 6.92–35.26%, elasticity between 63–70%, and H/D between 3.17–19.05%. The rheological profile of the dough obtained with lupin flours indicates a moderate stability and proper kneading behavior. The sensory analysis was also performed in order to identify the consumer’s acceptability regarding this type of bread. According to a 5-point hedonic scale, the most highly appreciated was the bread with 10% lupin flour, which obtained mean scores of 4.73 for general acceptability as compared with control bread (4.43).

## 1. Introduction

Lupin (*Lupinus* spp.) is a legume used as a raw material in food and feed production. Its many nutritional and health-promoting benefits have increased its popularity and consumption in recent years [1,2].

Lupin is cultivated in the North American and South American continents as well as in the European and African areas of the Mediterranean basin as a food or as an ornamental plant. The top three lupin-producing countries in 2020 in the world were Australia (474.6 metric kilotons), Poland (261.5 metric kilotons), and Russia (103.8 metric kilotons) [3]. For comparison, global wheat production in the same year was 760 million tons (top producers China, India, and Russia) [4].

Of the more than 200 species, in addition to all wild varieties, there are species cultivated for human consumption, others as ornamental plants, and most as animal feed. The progress of genetics in the twentieth century has contributed to the complete domestication of lupin species by hybridizing those with weak alkaloids and those with soft seeds, giving rise to new varieties that are sweet and more suitable for human consumption [5].

Certain varieties of lupin (*Lupinus albus*, white lupin; *Lupinus luteus*, yellow lupin; *Lupinus angustifolius*, blue lupin) are used in food, especially in the Mediterranean area. Over the last 20 years, researchers have looked to use lupin as a substitute for soybeans in vegetable production, with Australia (globally) and Germany (EU level) being in first place. In these countries, lupin and soybeans are grown alternately [3]. 

With the consolidation of the gluten-free and vegan market segment in healthy eating, sweet lupin grains have begun to replace soy in food (pasta, flour, tofu). In terms of nutritional benefits, lupin seeds are an alternative to soy, as they are non-genetically modified organisms. Especially in this second direction of exploitation, namely in healthy food, sweet lupin can be a solution to increase income for owners of small areas that are dedicated to organic farming [6].

The proximate composition of raw lupin depends by genetic and agronomical factors, and the composition of lupin can be affected by water stress; e.g., lipid content was reduced by half in conditions of water stress [7]. The lipid fraction ranges from 5.5 g/100 g dw in *L. luteus* to 18.9 g/100 g dw in *L. mutabilis*. Lupin is rich in in protein (up to 39%), with a protein content four times higher than in wheat grains. It also has one of the highest fiber concentrations (32%) and contains a very small content of starch. Lupin does not contain cholesterol [8] or gluten and gastric irritants (unlike soy, which has a fairly high amount of saponins). Corresponding to its low starch content, it has a very low glycemic index (GI) and is indicated for people with diabetes or who want to lose weight [8]. Lupin is probiotic, helping to grow good bacteria in the body, and contains an appreciable amount of essential amino acids. Lupin contains substantial amounts of polyphenols, carotenoids, phytosterols, tocopherols, and peptides. The plant is also known for its therapeutic properties due to its antioxidant, antimicrobial, anticarcinogenic, and anti-inflammatory activities [9,10].

Lupin is processed into various forms, as it is included in bread (which gives it a very pleasant taste) and various pastries but also in meat, sausages, and drinks, and its use is expected to increase significantly in the coming years as resources become more limited. Lupin berries contain proteins that can be used to produce meat and dairy substitutes, as they are suitable for all requirements for use by vegetarians and more.

Lupin flour might be integrated in bread formula in different forms: dehulled or hulled flour, hydrolysates or protein isolates, germinated or fermented flour, and as single- or multi-pulses blends [11]. The addition of lupin in flour composites has positive nutritional, chemical, physical, and functional impact. In addition, pulses flours are recognized for their high water-holding capacity, solubility, foaming, gelling, and emulsifying properties that influence the rheological behavior of the dough [12].

Although the literature [13] suggests that lupin grain has a partial allergy risk, the requests of such products are increasing in specialty stores. Sales of these products indicate that only a very small percentage of consumers are affected by potential allergens. In articles published recently, lupin flour addition ranged from 5–20% [14] and from 0–30% [15]. The aim of this paper is to obtain lupin/wheat flour composites and three lupin/wheat breads, which were characterized from a nutritional, phytochemical, technological, textural and sensory point of view in order to use these food products as a sustainable and functional food in a healthy diet. 

## 2. Materials and Methods

### 2.1. Preparation of Flour Composites 

Lupin organic flour (LF) produced by Dried Fruits Suppliers was purchased from VitaMix40 shop in Timisoara, Romania, and was obtained from the sweet lupin seeds variety *Lupinus albus*. Wheat flour (WF) type 550 was purchased from Auchan supermarket in Timisoara, Romania. Based on the literature studies [7] and our own experience on obtaining flour composites [16,17,18,19], three types of flour composites were obtained: WFL10 (10% lupin flour and 90% wheat flour); WFL20 (20% lupin flour and 80% wheat flour); and WFL30 (30% lupin flour and 70% wheat flour).

### 2.2. Determination of Proximate Composition of Flours Mixes

The proximate composition was determined in accordance with guideline of the Association of Official Analytical Chemists (AOAC 2000) standard method [20]. The total carbohydrate content (%) was determined as the difference between 100 and the sum of the following fractions: proteins, lipids, ash, and moisture.

*Carbohydrates* (%) = 100 − (*lipids* + *proteins* + *water*) × 100 (1)

By summing up the caloric intake produced by the individual nutrients (lipids, carbohydrates, and proteins), the energy value of the products was determined taking into account the following correlations: 1 g lipids = 9 kcal, 1 g carbohydrates = 4 kcal, and 1 g proteins = 4 kcal.

*Energy value* (*kcal*/100 *g*) = (*lipids* × 9) + (*carbohydrates* × 4) + (*proteins* × 4) (2)

### 2.3. Phytochemical Profile of Lupin Flour Composites and Bread

#### 2.3.1. Preparation of the Alcoholic Extract

From each sample, 1 g was weighed and placed in containers lids over which 10 mL of 70% ethanol (SC CHIMREACTIV SRL, Bucharest, Romania) was added. The containers were closed and shaken for 30 min using the hot plate shaker (IDL, Freising, Germany). After stirring, the extracts were filtered through a Whatman filter paper (Whatman, Maidstone, UK).

#### 2.3.2. Assessment of Total Phenolic Content (TPC)

The total phenolic content (TPC) of flour composites and breads with different percentages of lupin flour was analyzed according to the modified Folin–Ciocâlteu method [21]. The results were expressed in mg gallic acid equivalent (GAE) per 100 g of samples. The calibration curve was established in the range of concentrations of 2.5–250 μg/mL. All determinations were made in triplicate.

#### 2.3.3. Determination of Total Flavonoids Content (TFC)

The method presented by [22] was used for total flavonoids content (TPC). The results were expressed as mg quercetin (QE) per 100 g of samples, and all determinations were performed in triplicate. The calibration curve was obtained with quercetin (concentration range: 0.5–50 μg/mL).

#### 2.3.4. Antioxidant Capacity Determined by DPPH (1,1-diphenyl-2- picrylhydrazyl) Radical Scavenging Activity 

In order to determine the antioxidant activity of flour composites and bread, the method described by Ciulca et al. (2021) [23] was used. Each sample was analyzed in triplicate. The antioxidant activity (AA) was calculated as a percentage of radical scavenging capacity according to the following equation:

AA (%) = [(A_control_ − A_sample_)/A_control_] × 100 (3)

where A_control_ and A_sample_ are the absorbance values of the control and the tested samples, respectively.

### 2.4. Assessment of Macro and Microelements Composition 

The assay of macro and microelements content both for flour composites and bread was carried out by atomic absorption spectroscopy (AAS) using Varian 220 FAA equipment after calcination of the samples. Briefly, 3 g of each sample were burned using calcination furnace (Nabertherm GmbH, Lilienthal, Germany), at a temperature of 650 °C. The white ash obtained was dissolved in HCl 20% and further used to determine macro- and microelements. For calibration curve, a mix standard solution (ICP Multi Element Standard solution IV CertiPUR) was used. The minimum detection limits (MDL) for analyzed elements were 0.02 ppm for Mg and K; 0.06 ppm for Fe, Cu, Zn, and Mn; and 0.03 ppm for Ca. The results were expressed in ppm. All determinations were made in triplicate [24].

### 2.5. Assessment of Fatty Acid Composition 

Fatty acid profile was determined after derivatization as fatty acid methyl esters (FAME) according the method described by Posta et al. (2022) [24].

Fatty acids were identified based on the NIST 05 spectrum library using peak area normalization method by relating the peak area corresponding to a given compound to the total area of all peaks. All the analyses were conducted in three replicates. Saturated fatty acids (SFA) were calculated based on the sum of C4:0–C24:0, monounsaturated fatty acids (MUFA) were calculated as sum of C16:1–C22:1, and polyunsaturated fatty acids (PUFA) were calculated as sum of C18:2, C18:3, and C20:4. Unsaturated fatty acids (UFA) were calculated as sum of MUFA and PUFA.

#### Calculation of Atherogenic Index (AI) and Thrombogenic Index (TI)

On the basis of the identified fatty acids, the atherogenic index (AI) and the thrombogenic index (TI) were calculated using the following equations [8]:

AI = [C121:0 + (4 × C14:0) + C16:0]/[(n-6PUFA) + (n-3PUFA) + MUFA](4)

TI = (C12:0 + C16:0 + C18:0)/[(0.5 × MUFA) + (0.5 × n-PUFA) + (3 × n-3PUFA) + [(n-3PUFA)/(n-6PUFA)](5)

### 2.6. The Technological Process to Obtain Lupin Bread

**Figure 1:**
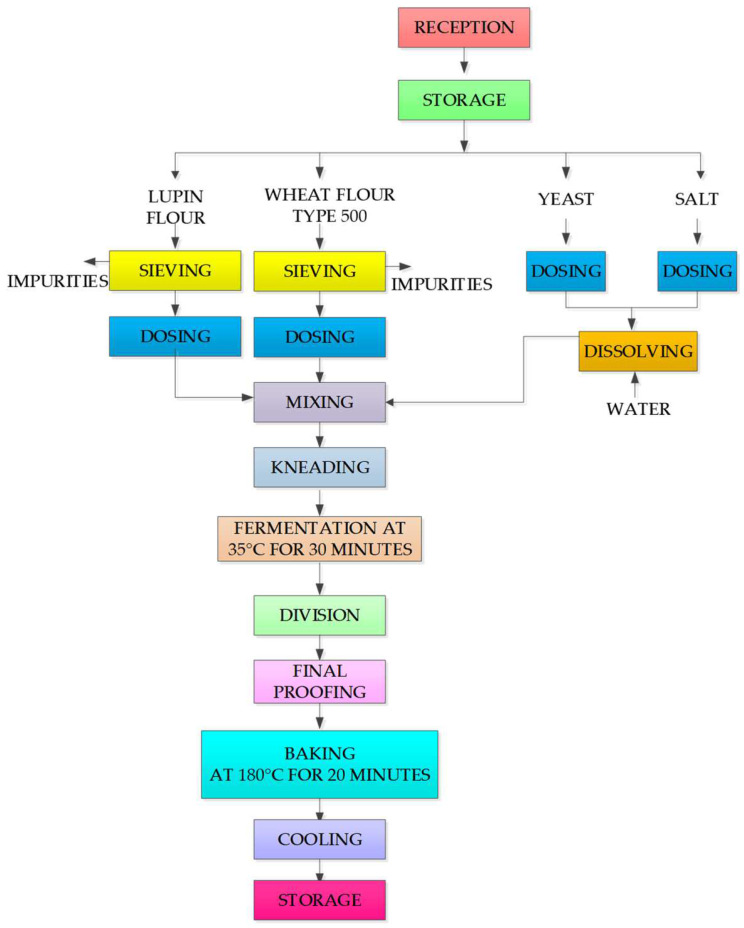
The technological chart-flow for obtaining bread.

**Figure 2:**
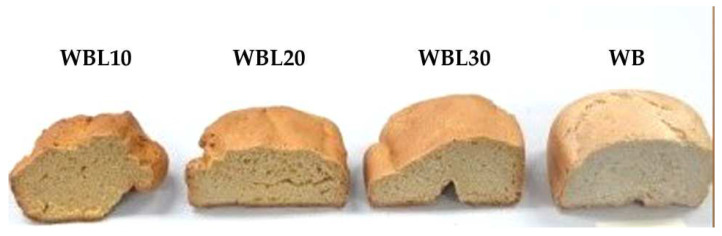
Final products. WB, wheat bread; WBL10, lupin/wheat bread 10%; WBL20, lupin/wheat bread 20%; WBL30, lupin/wheat bread 30%.

Figure 1 shows the technological chart-flow for obtaining bread, the technological phases, and working parameters for each stage. The technological process was performed according to the method described by Alexa E. (2008) [25]. The bread with lupin composite flour was obtained in 4 experimental variants, namely WBL10 (lupin/wheat bread 10%), WBL20 (lupin/wheat bread 20%), WBL30 (lupin/wheat bread 30%), and WB (wheat bread) (Figure 2), according to the manufacturing recipe presented in Table 1. In order to highlight the advantages and/or disadvantages of fortifying classical bread with lupin flour, a control sample (WB) made exclusively from wheat flour was also included in the study, following the same protocol in the manufacturing process. 

### 2.7. Physical-Chemical Properties of Lupin Bread

The quality parameters such as moisture content, acidity, volume, porosity, springiness (elasticity of core), and the height/diameter ratio were determined according with SR 91:2007 [26]. The results are expressed as the arithmetic mean of the two parallel determinations.

### 2.8. Rheological Properties

Rheological and sensory properties are of major importance in the acceptability of a novel food product by consumers. The rheological properties of wheat and lupin flours and also of flour mixes flours were analyzed using Chopin Mixolab test (Chopin Technologies, Paris, France) using the “Chopin+” protocol [27]. 

A quantity of 50 g of sample was placed into the Mixolab bowl and mixed. After mixing the solid part, the necessary water was added by the equipment to obtain an optimal consistency. During the tests, the running parameters of the Mixolab equipment are:

- 80 rpm mixing rate;
- First plateau: time 8 min, temperature 30 °C, gradient 4 °C/min;
- Second plateau: 7 min, temperature 90 °C, gradient 4 °C/min;
- Third plateau 50 °C, 5 min [28].

The parameters evaluated from the Mixolab profile were: water absorption; dough development time; stability (mixing resistance of dough); maximum torque during mixing (C1); weakening of the protein (C2), which appears due to mechanical stress as the temperature rises; rate of starch gelatinization (C3); minimum torque during the heating period (C4); and torque after cooling at 50 °C (C5). Other parameters determined by Mixolab were protein-weakening speed under heating effect (alpha slope); starch-gelatinization speed (beta slope); enzyme-degradation speed (gamma slope); cooking stability (C4/C3); and starch retrogradation at cooling stage (C5–C4), which represents the shelf-life of the final products [29].

### 2.9. Sensorial Analysis of Bread 

Bread samples WBL10, WBL20, and WBL30 were evaluated by a panel consisting in 44 untrained assessors (20 males and 24 females) between the ages of 19 and 52, non-smokers, ad without known cases of food allergies. The slices of bread, with the crust, were presented (1 cm thick) on cardboard plates, encoded in double-digit characters, and served in random order under normal lighting conditions and at room temperature. To evaluate the consumer’s acceptance, a five-point hedonic scale was used [30], with the following rates: 1 = extremely dislike; 2 = slightly dislike; 3 = neither like nor dislike; 4 = slightly like; and 5 = extremely like.

Panelists were asked to evaluate the sensory attributes of the samples, including appearance, color, texture, flavor, taste, and general acceptability based on their liking degree. The ranges of score and acceptability level were as follows: 1.00–1.49 = not acceptable (NA); 1.50–2.49 = slightly acceptable (SA); 2.50–3.49 = moderately acceptable (MA); 3.50–4.49 = acceptable (A); and 4.50–5.00 = highly acceptable (HA) [30]. 

### 2.10. Statistical Analysis

All determinations were made twice or in triplicate depending on the method, as is presented in each subchapter of materials and methods, and the results are reported as mean values ± standard deviation (SD). Differences between means were analyzed with a one-way ANOVA, followed by multiple comparison analysis using the *t*-test (two-sample assuming equal variances) using Microsoft Excel 365. Differences were considered significant when *p*-values < 0.05. 

## 3. Results and Discussion

### 3.1. The Proximate Composition of Lupin Flour Composites and Bread

The results presented in Table 2 illustrate the proximate composition of lupin flour composites, lupin, and breads.

It is observed that there are significant differences in the protein, lipid, and mineral content of lupin flour (LF) and wheat flour (WF) that are reflected in the proximate composition of mixed flours (WFL). LF, like other legumes, is characterized by a significant protein intake (30.95%) compared to WF (12%), lipid content (12.42% LF and 0.32% WF), and macro- and microelements intake (3.67% LF compared to 0.66% WF), which recommends LF as a functional matrix in baking. The moisture level of the samples falls within the same range of values, without significant differences recorded for the flour composites. The carbohydrate content was lower in the LF sample (45.7988%) compared to WF (80.105%), contributing to the decrease of the carbohydrate content of flour composites even with a minimal addition of LF in WF.

The increase in protein fraction by addition of LF in WF varies between 14.67–52.92% in flour composites and between 13.10–58.75% in composite breads, respectively. In the same way, the lipid content increased between 418.75–1156.25% in flour composites reported with the initial content in WF and between 170.59–185.29% in bread composites compared with white wheat bread.

The bread composites variants (WLB) are distinguished by a protein, lipid, and mineral content superior to the control bread, and these hardly increased with the proportion of LF added to the WF. Significant differences in protein content are noted in the case of the addition of 10 to 30% LF in bread composition. The lipid content is tripled by the substitution of WF with 10–30% LF, but there are no significant differences in the case of composite bread variants (WFL10–WFL30 and WBL10). The mineral substances in the bread obtained with flour composites increase with the addition of LF, with significant differences compared to the control bread being registered in the case of the addition of 20 and 30% LF. However, the carbohydrate content is significantly lower in the case of bread with flour composites.

Lupin flour is widely used in various foods in bakery and confectionery products, protein concentrates, and other industrial products due to its nutritional quality and its high functional properties [31,32]. The functional properties of flours obtained from different lupin species, and its applications in foods have been previously reported [2,33,34]. Kefale B. and Yetenayet B. (2020) [35] reported for LF a content of 7% moisture, 4.2% mineral substances, 35.08% protein, and 7.65% lipids. Moreover, LF has been reported to contain large amounts of soluble and insoluble dietary fiber fractions [36]. 

Our study aimed to obtain three prototypes of functional bread with the addition of 10%, 20%, and 30% lupin flour. The comparative analysis was carried out to explain the impact of different proportions of LP added to the WF on the proximate composition of breads. 

The bread samples studied had humidity content between 32.86% and 37.92%. Similar results were reported by Wandersleben et al. (2018) [11] for lupin bread, which reported a moisture content of 35.00% compared to the moisture content of the control sample of 37.00%. 

Vegetable proteins are increasingly sought after lately due to their content in essential amino acids, which are especially important for a balanced diet. Since WF is low in protein [37], LF supplementation is beneficial for increasing the nutritional value of the products to which it is added. Bread with the addition of LF comes with a significant intake of high-quality protein, showing increases from 7.71% in the case of WB, to 8.72% in WBL20, 10.41% for WBL20, and 12.24% for WBL30. Similar results for bread with the addition of 15% of LF were reported by Wandersleben et al. (2018) [11], namely a percentage of 11.1%. Increases in protein content were also reported by Serventi et al. (2018) [38] for chickpea bread and Previtali et al. (2014) [39] for bread with lentils. Kefale B. and Yetenayet B. [35] studied bread with different proportions of LF and reported a proportional increase in protein content with the added concentration of LF.

In addition to a significant increase in protein content, bread with the addition of LF provides a significant intake of lipids. There was an increase from 0.34% in the case of the WB samples: 0.92% for WB1, 0.94% for WB2, and 0.97% for WB3.

A trend of increasing lipid content for bread with the addition of LF was also reported by Previtali et al. (2014) [39] and similar lipid content for lupin bread (1.1%) obtained by Wandersleben et al. (2018) [11].

Our results show that the ash content in the samples of lupin flour bread slightly increased from 0.74% for WB to 0.77% for WB1, 1.15% for WB2, and 1.34% for WB3. This increase is due to the relatively high content of mineral substances in lupin flour (3.67%). Similar results were mentioned by Wandersleben et al. (2018) [11], who reported a content of 1.50% ash for lupin bread. Significant increases in ash and lipid content in bread with the addition of lupin flour were also reported by Alomari et al. (2013) [7].

The carbohydrate content recorded was 52.34% for WB, the values decreasing with the increase in the proportion of lupin flour as follows: 52.21% for WBL10, 51.73% for WBL20, and 51.44% for WBL30. A lower carbohydrate content in lupin bread was also reported by Wandersleben et al. (2018) [11] in their study, namely 43.60% compared to 51.00% in wheat flour bread.

Another advantage of adding LF to bread is to increase the energy value from 243.23 kcal/100 g for WB to 251.99 kcal/100 g for WBL10, 250.13 kcal/100 g for WBL20, and 242.48 kcal/100 g for WBL30 without increasing the carbohydrate content. The same trend was reported in the case of fortification of bread with hemp flour [16].

### 3.2. Phytochemical Profile of Lupin Flour Composites and Bread

Phytochemical profiles of lupin flour composites and bread (TPC, TFC, and AA) are presented in the Table 3. 

The obtained results shown that the content of total polyphenols (TPC) increased with the level of LF added in the composite samples. The TPC content is more than double in LF compared to WF, with significant differences regarding this parameter being recorded between the flour composites and breads.

The recorded increases in flour composites compared to the WF varied between 5.58–58.99% for TPC, between 15.48–56.99% for TFC, and between 32.88–55.47% for AA. The same pattern was highlighted when wheat bread was fortified with LF. The increases of functional parameters in the bread composites compared with WB were 35.86–140.64% for TPC, 33.02–166.04% for TFC, and 15.60–79.03% for AA.

It is also worth noting that the TPC content in bread samples is lower than the TPC content in flour composites. Thus, in the case of flour samples, it can be seen that the highest TPC content was found in WFL30 (85.41 ± 1.94 mg GAE/100 g sample), while the lowest value was recorded for the WF control sample (53.72 ± 0.38 mg GAE/100 g). In the case of bread samples, the highest TPC content was found in WBL30 (76.50 ± 0.70 mg GAE/100 g sample), while the lowest value was recorded for the WB control sample (31.79 ± 0.58 mg GAE/100 g). 

The total flavonoid content (TFC) showed the same pattern as the TPC profile. Hence, the TFC was maximal in the LF and minimal in the WF. Furthermore, the TFC recorded in the bread samples was lower than the TFC recorded in the flour samples. Thus, in the case of flour composites, the TFC was within the limits of 4.65 ± 0.07 mg QE/100 g and 12.36 ± 0.11 mg QE/100 g, with the highest value being found in LF, while the lowest value was recorded for the WF control sample. In the case of flour mixtures, the TFC content increased in a statistically significant way with addition of the LF content due to the high content of flavonoids in lupin flour.

In the case of bread samples, the TFC content varied within 3.21 ± 0.06 in WB and 8.54 ± 0.19 mg QE/100 g in WBL30. Similar with the TPC profile, the TFC decreased in the case of bread samples compared to the flour samples from which they were prepared. Statistically significant differences were recorded within the TFC content of bread samples with a different percentage of lupin.

The same trend measured for TPC and TFC was maintained in the antioxidant activity (AA) profile. In the case of flour samples, the AA values were within 52.98 ± 0.24% and 93.37 ± 1.53%. The highest value was recorded for LF (93.37 ± 1.53%) and the lowest value for WF (52.98 ± 0.24%). In the case of flour composites, the AA increased with the percentage of LF added and is correlated with the high AA of LF (data not shown). Statistically significant differences were detected within all composite and non-flour composites.

In the case of bread samples, the AA was within the limits of 30.71 ± 3.82 and 54.98 ± 1.21%, the highest value being measured for WBL and the lowest value for the WB control sample. As TPC and TFC profiles, the AA decreased in bread samples compared to the flour samples from which they were prepared.

Lupin flour is a good source of polyphenols, and this is due to the high content of polyphenols reported in lupin seeds [9]. Several studies have highlighted the phytochemical value of lupin seeds and flour [40,41,42,43,44,45,46,47].

Dalaram (2017) [40] reported that in dry matter of lupin seeds, the TPC varies between 367.36 mg GAE/100 g and 696.21 mg GAE/100 g depending on the variety, while Liezhou Zhong (2021) [41] found lower values (36.11 ± 0.31 mg GAE/100 g). Karamać et al. (2018) [42] reported a total polyphenolic content of lupin seeds between 4.36 to 7.24 mg GAE/g dry matter (d.m.). Vollmannova et al. (2021) [43] studied the TPC in several varieties of lupins and found a content between 4260–5663 mg GAE/kg. Other authors reported 14.23 ± 1.38 μg GAE/g [44], 313.9–744.7 μg GAE/g [45], and 1294 μg GAE/g [46], respectively. The values obtained are varied and depend on the source of lupin seeds, cultivation, or processing conditions. 

The TPC content of analyzed breads with different levels of LF varies between 43.19 and 76.49 mg GAE/100 g. The study regarding the improvement of functional cake formulation with fermented lupin powders highlighted that the TPC decreased by addition of LF and varies between 220–264 mg GAE/100 g in cakes depending on the content of LF. The same author reported a TPC content in fermented lupin powder of 298 mg GAE/100 g [47]. The higher values reported in this study are probably due to the fermentation of LF, which leads to a better bioavailability of the active principles.

The TFC varies in flour composites between 46.50–73.03 μg/g and between 42.70–85.41 μg/g in composite breads. Results in the same range of values were reported by other authors: 12.40–16.60 μg/g [44] and 108.8–376.1 μg/g [45], respectively. The antioxidant activity of LF (93.375%) corresponds with the data from the specialized literature: 93.79% [44] or 83.6% [48].

The processing of lupin seeds influences the phytochemical profile. During debittering, the content of tocopherols and bound flavonoids slightly increased, while free phenolics and bound phenolic acids decreased by 76.2% and 50.1%, respectively. The extrusion of lupin seeds slightly increased TPC, but the spray-drying process diminished TPC [49].

### 3.3. Macro and Microelements Profile of Lupin Flour Composites and Bread

In Table 4 the macro- and microelements content of lupin flour composites and breads are presented. 

Magnesium (Mg) is the most abundant macroelement in the analyzed samples and varies between 124.67 ± 5.66 mg/kg in WB and 783.33 ± 14.14 mg/kg in LF. Statistically significant differences were found both in terms of Mg content in lupin, wheat, and flour composites but also in the bread obtained.

Magnesium is a mineral that plays an essential role in the optimal functioning of the body. More than 600 enzymatic reactions depend on it, including energy metabolism and protein synthesis. Magnesium is needed for calcium and potassium ion transport, muscle contraction and relaxation, neurotransmitter release, protein biosynthesis, cellular energy production, mitochondrial function, cell membrane stabilization, transmembrane ion flow, genome stability, bone development, and glucose homeostasis. The National Institutes of Health recommends that the daily magnesium intake of the healthy population be over 400 mg for men and over 310 mg for women depending on age.

Our results show that the addition of LF (Mg content 78.3 mg/100 g) in the flour composites and breads leads to the enhancing of Mg content, with the maximal content of Mg being recorded in the WFL30 and WBL30 samples. Similar results were reported by other authors regarding the content of Mg in LF: 33.93 mg/100 g [50], 82.61 mg/100 g [47], and 34.49 mg/100 g in the bread with 20% lupin and 22.41 mg/100 g in the cake with 30% LF.

The calcium content (Ca) in the WFL10 (226.00 ± 6.36 mg/kg) and WFL20 (244.64 ± 5.65 mg/kg) composite flour samples did not show significant differences from WF (225.33 ± 6.60 mg/kg). The LF recorded the highest Ca content (290.00 ± 7.07 mg/kg) without significant differences related to WFL30 sample (287.67 ± 6.48 mg/kg). WB content was 129.00 ± 4.24 mg/kg, lower than the WBL that recorded values between 262.00 ± 5.66 mg/kg (WBL10) and 533.00 ± 10.61 mg/kg (WBL30).

Calcium plays an essential role in the human body, ensuring the normal functioning of the osteoarticular system, bone density, and the functioning of muscle and nerve cells. A vital macro-element for humans, calcium is found in many foods, but its fixation in bones also depends on the presence of other vitamins and minerals.

Our results regarding the Ca content in flour composites and breads are in agreement with previous studies that reported 37.69 and 38.15 mg Ca/100 g lupin flour depending on the flour type [51]. Additionally, in bread obtained with ultrasonicated LF, the level of Ca varied between 29.24–44.42 mg/100 g depending of the percentage of LF added in the bread formula and the method used for debittering of LF [50]. Other study reported 99.04 mg/100 g Ca in cakes fortified with LF [47].

Potassium content (K) in flour composites varied in the range 70.32 ± 1.41 mg/kg and 165.33 ± 5.66 mg/kg and increased with LF addition. Significant differences were observed between WB (162.33 ± 4.24 mg/kg) and composite breads, but no significant statistical differences were recorded between WBL10/WBL30.

Potassium is an essential element for the balance of electrolytes and body fluids. It is also the third most present mineral in the body and is needed for the functioning of many organs, including the heart, kidneys, brain, and muscle tissue. The content of K in LF was found by Aslan et al. (2019) [47] to be 53.65 mg/100 g and increased in cake sample at 179.35 mg/100 g when 30% LF was added in the recipe. The study of Yaver et al. (2021) [50] reported 221.5 mg K/100 g LF and highlighted that the K content of bread fortified with lupin flour decreased with the percentage of LF. The same pattern regarding K level was observed by the authors with the pasta obtained with LF [51].

The debittering process influences the content of K in LF. Therefore, K in bitter LF was found to be 980.55 mg/100 g, while in debittered LF, it was 23.15 and 27.82 mg/100 g depending of the process type [51]. In bread, the maximum K content was 203.35 mg/100 g corresponding to 30% LF added in the composition [50]. The LF used in our study was a commercial, debittered LF with a low K content of 16.50 mg/100 g.

The first studies on the importance of the presence of zinc in the diet appeared after the 1960s, when, in 1961, the clinical deficiency of zinc was described for people with a poor diet in products containing this mineral. An adult needs a maximum of 3 g of zinc daily. Over 60% of this amount is used to mineralize the bones that support the skeleton, 30% is used for the bones in the joints, and the remaining 10% is used in the metabolism of the liver and the structure of the epidermis. The Zn content in our samples was 1.78 mg/100 g for LF and 1.20 mg/100 g for WF. The addition of LF increased the content of Zn until 1.4 mg/100 g in bread with 30% FL. Similar results were obtained by other authors: 0.94 mg/100 g in LF and 1.16 mg/100 g in bread with 20%FL [50] and 1.29 mg/100 g in cake with 30% LF [47].

The copper (Cu) content determined in the analyzed flour samples was in the range of 3.21 ± 0.21 mg/kg and 3.46 ± 0.09 mg/kg and in the bread samples was in the range of 0.60 ± 0.01 mg/kg and 1.67 ± 0.06 mg/kg.

Copper is an essential microelement for the body; along with iron, it plays an important role in the formation of red blood cells and in maintaining the health of blood vessels, nerves, bones, and the immune system. Literature studies reported 0.23–0.71 mg/100 g Cu in LF [51], which is similar to the measurements obtained by us in LF (0.35 mg/100 g) and in flour composites (0.23–0.32 mg/100 g). The addition of LF in WF increased the level of Cu in bread obtained with flour composites. 

The copper level in the LF was found to be 3.46 ± 0.09 mg/kg but was not detectable in WF. The supplementation of WF with LF increased the content of Cu both in flour composites and breads.

Between analyzed microelements, manganese (Mn) recorded the highest level in LF (136.74 ± 4.99 mg/kg), while WF content was only 0.25 ± 0.001 mg/kg. The addition of LF in composite flour increase the level of Mn in bread, reaching the highest level in WBL30 (117.37 ± 6.55 mg/kg) compared to 2.95 ± 0.15 mg/kg in WB.

Manganese is a trace element that forms part of mitochondrial enzymes and also activates many enzymes. Thus, manganese plays an essential role in lipid and carbohydrate metabolism, bone tissue formation, and reproductive processes. A high content of Mn was found in the sample of LF analyzed in our study (13.60 mg/100 g) and low content in WF (0.25 mg/100 g). The addition of LF in bread enhanced the Mn content until the 11.70 mg/100 g level for the WBL30 sample. Further, 10.56 mg Mn/100 g was detected in cake obtained with 30% LF [46] and 12.47 mg/100 g in bread with 30% LF [50], respectively.

The highest level of iron (Fe) was recorded in LF (22.00 ± 1.41 mg/kg) compared with WF (0.51 ± 0.01 mg/kg). In composite bread, the level of Fe varied between 124.67 ± 5.66 mg/kg in WB and 403.00 ± 10.32 mg/kg in WBL30, with significant statistical differences between samples.

Iron is an essential mineral for the proper functioning of the body and for maintaining long-term health. According to statistics, about 20% of women, 50% of pregnant women, and 3% of men lack iron in the body, and deficiency leads to severe diseases, the most common being anemia. The level of Fe in analyzed samples varies between 2.09 mg/100 g in LF and 1.482 mg/100 g in WBL30. Similar values were reported in LF (6.12 mg/100 g), in bread with 20% LF (2.65 mg/100 g) [46], between 174–2.09 mg/100 g in bread with different percentage of LF [50], and between 4.35–6.15 mg/100 g in bitter LF and debittered LF using different methods [51].

Zinc (Zn) is the element that was found in higher quantities in WF (23.35 ± 0.50 mg/kg) compared with LF (12.79 ± 0.18 mg/kg).

Flour composites nickel (Ni) and chromium (Cr) is minor elements found in WF and LF, and their changes in the flour composites and breads do not influence the mineral composition. 

### 3.4. Fatty Acids Profile of Lupin Flour Composites and Bread

The fatty acid composition, SFA, and the UFA (monounsaturated MUFA plus PUFA) content for composite lupin flours and breads are reported in Table 5, Table 6 and Table 7. With the technical of GC-MS, 22 fatty acids were separated from the analyzed samples, of which 11 are saturated fatty acids, 8 are monounsaturated fatty acids, and 3 are polyunsaturated fatty acids.

From the analysis of the data obtained, LF has a lower proportion of saturated fatty acids (SFA), with a value of 32.92%, the predominance being palmitic acid (C16:0) (21.38%) and stearic acid (C18:0) (5.50%). The higher content is found in monounsaturated fatty acids (MUFA) (48.03%), predominantly being oleic acid C18:1n9 (40.58%), oleic (C18:1n7) (2.16%), and eicosenoic acid (C20:1n9) (3.52%). As for the polyunsaturated fatty acids (PUFA), they are found in a proportion of 18.95%, the most significant proportions belonging to linoleic acid (C18:2n6) (18.64%). In contrast, for WB, a higher content of SFA was recorded at 36.68%, the most predominant also being palmitic acid C16:0 (22.44%) and stearic C18:0 (12.20%). The proportion of MUFA in WB was significantly lower at 19.44%, predominantly being oleic acid C18:1n9 (17.14%) and eicosenoic acid (C20:1n9) (1.63%), but the proportion of PUFA was much higher (42.50%), and for WB, the predominant proportion was that of linoleic acid (C18:2n6) (40.92%). In terms of flour mixtures, the proportion of SFA is inversely proportional to the LF content, at 39.08% for WFL10 and 37.01% for WFL30. In contrast, the proportion of UFA is directly proportional to the LF content, namely 61.22% for WFL10 and 62.08% for WFL30.

As regards the bread samples, it can be noted that the samples containing LF had a lower content of saturated fatty acids compared to the control sample (64.40%), with this content being inversely proportional to the proportion of added LF (37.07% for WBL10 and 36.20% for WBL30 versus 40.49% for WB). Regarding the content of unsaturated fatty acids, their proportion increases with the increase in the content of added LF (62.92% for WBL10 and 63.43% for WBL30 compared to 59.36% for WB). This is due to the low content of SFA (32.95%) in lupin and the high content of MUFA (especially oleic acid C18:1 40.58%) compared to WB, which recorded a SFA content of 38.68% and MUFA content of 19.44%. 

Lupin has an interesting unsaturated fatty acid (FA) profile mainly consisting of oleic and linoleic acids and ratios between fatty acids, which confer upon it specific functionalities. Unsaturated fatty acids (UFAs) are essential nutrients, and the n-3/n-6 polyunsaturated fatty acids (PUFA) ratio is considered very important with respect to human and animal nutrition.

The similar composition of fatty acids in lupin flour with those detected in our study was also reported by Uauy et al. (1995) [52], who identified a palmitic acid content of 11.00%, stearic 5.50%, oleic 30.80%, linoleic 18.70%, and behenic 2.80%. Andrzejewska et al. (2016) [53] analyzed the fatty acid composition of white lupin and identified a palmitic acid content of 8.57%, stearic 1.57%, oleic 54.30%, linoleic of 14.90%, and erucic of 1.59%; in another study, Alamri (2012) [54] recorded a palmitic acid content of 7.71%, stearic 1.71%, oleic 44.90%, and linoleic 26.20%. For wheat flour, Zengin et al. (2017) [55] studied the fatty acid composition of seven wheat cultivars grown in Turkey and identified a similar content recorded in this paper, namely palmitic acid (C16:0) between 24.10 and 35.83%, stearic acid (C18:0) 1.64–5.16%, oleic (C18:1) 15.13–19.62%, and linoleic acid (C18:2) 29.52–39.58%. Similar values for wheat flour also recorded by Nikolic et al. (2008) [56] in his study, in which he recorded a content of 19.56% palmitic acid (C16:0), 1.37% stearic acid (C18:0), 20.28% oleic acid (C18:1), and 57.67% linoleic acid (C18:2%). A similar pattern was also reported by Rusu et al. (2021) [16], who tracked the influence of the addition of hemp flour in different proportions in white bread.

Fatty acids composition plays an important role in control of cardiovascular diseases. To evaluate the different effects of the fatty acids, two indices, atherogenic index (AI) and thrombogenic index (TI), were developed in order to characterize the atherogenic and thrombogenic potential of food based on total saturated fatty acids (SFA) or on the polyunsaturated/saturated fatty acids ratio (PUFA/SFA) [57,58,59]. Lower values of AI and TI are associated with low cardiovascular, atherogenic, and thrombogenic risk. 

The AI and TI values presented in the Table 7 show that LF has lower values in terms of two parameters (AI: 0.40, TI:0.84) compared with WF (AI: 0.44, TI:1.02), composite bread flours (WBL), and wheat bread (WB), respectively. The AI index decreased with the addition of LF in WBL, and the lower value was obtained for WBL30 (0.42) compared with WB (0.46). The same pattern was recorded for the TI index (1.18 for WB and 0.97 for WBL30).

Similar values were reported by Chiofalo et al. (2012) [8] regarding different varieties of lupin species cultivated in a Mediterranean environment.

### 3.5. Physical-Chemical Proprieties of Lupin Bread

After baking, the volume, porosity, elasticity, height/diameter ratio (H/D), acidity, and humidity were determined for all samples according to SR 91:2007 [26]. The experimental results obtained are shown in Table 8.

Analyzing the data obtained, it can be pointed out that the volume, porosity, elasticity, and H/D index were higher in the case of the control sample than composite bread sample with different proportions of lupin flour; however, the products were not excessively flattened (Table 8). The decrease of volume compared to WB ranged between 0.69–7.37%, porosity between 6.92–35.26%, elasticity between 63–70%, and H/D between 3.17–19.05%.

The control sample (wheat flour, with no lupin addition) registered the highest values in terms of volume (434 cm^3^/100 g) and H/D ratio (0.632) compared to the samples with LF, which registered lower values regarding these parameters.

Bread with 10% lupin flour (WBL10) is well-leavened with a volume of 431 cm^3^/100 g, with soft and flat pores (61.08 % porosity) and an elastic core, and when it is pressed, it returns to it initial shape with 70% elasticity, and the H/D ratio is 0.613. Analyzing the porosity, it was found that in the case of the addition of lupin flour in a proportion of 10%, the appearance of the pores is maintained. 

The data showed that by adding LF, the bread H/D ratio decreases, the control sample shows a 0.632 ratio, and the samples with the three proportions of lupin flour recorded an H/D ratio between 0.51 (WBL30) and 0.61 (WBL10) (Table 6). For values of the H/D ratio between 0.4–0.6, it is considered that the volume and shape of the bread are appropriate, over 0.50 when the bread is bulging, and a value of the H/D ratio below 0.40 is an unsuitable, flattened product [26]. 

The data show that the volume and H/D ratio of all samples with the addition of LF were lower than that of the control sample (WB), but the porosity was more pronounced and uniform, the elasticity was better in the sample with 10% lupin flour (WFL10.), and the crust showed a more developed coloration due to the Maillard compounds formed during baking.

From the data presented in Table 8, it can be highlighted that the low volume of the composite breads may be due to the reduced amount of gluten from lupin, which may reduce its viscous–elastic properties and the dough’s capacity of gas retention during baking [60]. This effect of reduced volume of bread samples with LF has been reported by other researchers, too [61].

Figure 2 shows that the addition of lupin flour leads to defects in the bread: mainly shape and core defects, which become more obvious as the percentage of lupin flour increases.

The compact layers in the core, also known as “bacon layers”, look like a hard, non-porous, sticky mass. The layers appear in the form of horizontal stripes (WBL20) and can have different sizes. Compact layers appear in the center of the core because some of the water vapor formed in the peripheral areas suddenly enters the center due to the high temperature of the oven.

Another defect that is observed in the case of bread with the addition of lupin flour is the cracks in the crust and its irregular appearance (Figure 2).

The cracks may be on the surface of the shell or appear laterally. In all cases, they are formed due to the exit of fermentation gases from the dough, namely:

- When the flours come from sprouted grains or contain too little gluten; the bread crust is less elastic; and the fermentation gases, not meeting sufficient resistance to pressure, come out of the dough and form cracks on the surface of the bread;
- When the dough has an increased action of proteolytic enzymes, the resistance of gluten decreases, and the fermentation gases form a large number of small cracks, which greatly degrade the appearance of the bread [25].

### 3.6. Rheological Profile of Lupin Flour Composites

The primary parameters [27] of the mixtures with different proportions of LF are presented in Table 9. 

In Table 10 are presented the scores of the index defined by Mixolab profiler: water absorption index (WAI), mixing index (MI), gluten+ index (GI), maximum viscosity during heating expressed as viscosity index (VI), starch stability or amylolysis index (AI), and starch retrogradation–retrogradation index (RI).

The results presented in Table 9 show that water absorption (WA) increased by the addition of LF due to the lower humidity. WA increases from 54.80% in the case of WF to 60% at the addition of 10% LF in the composite mixture and up to 62.20% in the case of the variant with 30% LF. According to literature studies, the water absorption capacity in wheat flour ranges between 50–55% [25] and in composite lupin flours ranges between 71.3–84.1% [15]. Instead, the stability of the dough decreased with the addition of LF in the flour composites. Thus, if the dough obtained from WF has a stability of 9.52 min, it decreases as the proportion of LF in the manufacturing recipe increases from 6.62 min (WFL10) to 5.68 min (WFL20) and 5.47 min for WFL30. A similar pattern was observed by Hruskova et al. (2009) [15], who reported that a shorter dough stability and dough resistance compared with control were observed in composite flours. Doxastakis et al. (2002) [14] observed lupin’s technologically positive features, such as reducing mixing time, increasing water-holding capacity, and providing a distinctive flavor and yellow color of final products. The difference between the maximum torque at 30 °C and the torque at the end of the holding time at 30 °C (C1) is at a maximum in WF (1.189 Nm) and a minimum (1.122 Nm) in WFL20. 

Table 10 shows the Profiler Mixolab indices expressed on a scale from 0–9 (Mixolab Index) for a complete characterization of the flour (water absorption, mixing behavior, gluten protein / strength, hot viscosity, amylase activity, and relegation).

**Water absorption index (WAI)** depends on the quantity and quality of the flour components (protein, starch, fiber). Higher index provides a higher water absorption capacity. The absorption index is 2 for WF, 7 for WFL10, and increases to 8 with the addition of LF in the bread recipe. Similar values for wheat flour (WAI = 1) were reported by Antanas et al. (2013) [62], who highlighted that the addition of other matrices in flour composites increases the WAI index. 

**Mixing index (MI).** This index gives information on the behavior of the flour when it is kneaded at 30 °C and expresses the dough’s stability and softness. When this index increases, the dough becomes more stable at kneading. Our results are in line with previous study [62], namely that the maximum mixing behavior is obtained in the case of WF (MI = 6) and the minimum for the composite lupin–wheat breads (MI = 2), indicating a higher stability of the WF dough as WFL dough.

**Gluten+ index (GI)** measures the behavior of gluten during the heating of the dough. The higher the index, the more resistant the gluten is to the conditions of the technological process. Gluten resistance is maximum in WFL10 (5) and decreases with the addition of LF in the flour composites. WF has a lower index than WFL10. Similar results were obtained for flour composites from WF and triticale and rye flour [62]. 

**Viscosity index (VI).** The increase in viscosity during this phase is due to both the amylase activity and the quality of the starch. The higher the index, the higher it is the viscosity of the dough during the heat treatment. The viscosity of the dough is high for WF and decreases with the addition of LF in all analyzed samples (until index 1 for WFL30), which are values that give good dough-processing conditions. A previous study pointed out that the addition of 20% triticale flour in wheat flour decrease the VI from 7 (WF) to 6 in flour composites [62]. 

**Amylolysis index (AmI).** This index depends on the resistance of the dough to diastatic activity. The higher the index, the lower is the amylase activity. Amylase activity is maximal (8) in WF, while for WFL10, there is an index of 7, and for 20 and 30% LF in the composite dough, the AI is 6. WFL30 is distinguished by an increased amylase activity compared to other samples. The same value (7 for AmI) was reported for wheat flour, and a decrease of this index in flour composites is based on rye and triticale flours [62].

**Retrogradation index (RI).** Downgrade is due to the behavior of starch during cooling (crystallization of amylopectin). The higher the index, the shorter the expiration dates. RI for WF is 8 and decreases to 6 with the addition of LF in composite bread. This indicator provides valuable information on the shelf life of lupin bread, which can be kept longer without impairing physico-chemical indices, which gives it economic sustainability.

According to the Mixolab profile, five different stages regarding the dough torque depending on the time are presented in the diagrams (Supplementary File). The initial mixing period, when the temperature was constant, corresponds to the hydration of the compounds and occurs together with the stretching and alignment of the proteins, bringing about the formation of a three-dimensional viscoelastic structure [27].

The second stage of the Mixolab profile represents the first stage of heating and corresponds to protein weakening. The combined effect of mechanical stress and temperature induced a decrease in C2 torque, which is the minimum value of torque produced by the passage of dough subjected to mechanical and thermal constraints [10]. Compared to WF (C2 = 0.54), the flour composites with lupin are characterized by a lower C2 torque (0.41–0.46). The addition of LF in different percentages in WF leads to a decrease in C2.

In the third stage, starch gelatinization occurs. Heating the dough induces the swelling of the starch particles, and the viscosity of the dough increases. Cooking stage describes starch behavior and is characterized by following parameters: (i) maximum torque produced during the heating stage (C3); (ii) C4 calculated as a ratio of the torque after the holding time at 90 °C and the maximum torque during heating period; (iii) gelatinization rate (β); and (iv) cooking stability rate (γ) [27].

Torque point C3 expresses the viscosity of the dough on heating. The increase of this parameter highlights the increase of the viscosity of the dough. C4 and β value indicate the content of alpha-amylase in the flour, with the increase of this enzyme producing the increase of the two mentioned parameters [27]. The obtained results show the decrease of C3 and C4 values by the addition of FL in the flour composites, with the maximum values being registered in the case of WF (C3 = 2.03; C4 = 1.86) and the lowest values in the case of WFL30 (C3 = 1.27; C4 = 1.42), which highlights the decrease in the viscosity of the dough and the intake of alpha amylase by the addition of LF in WF.

In the fourth stage of Mixolab profile, the temperature is constantly maintained. During this phase, the dough suffers the double action of mixing and enzymatic attack, observed by a decrease of consistency.

The dough cooling (the fifth stage) leads to starch retrogradation, and the stiffness of the cooked dough increases. C5 represents the difference between the torque produced after cooling at 50 °C and heating period. C5 varies between 3.29–2.30, which is at the maximum in case of WF and minimum in WFL30.

### 3.7. Sensorial Analysis of Composite Lupin Bread 

**Figure 3:**
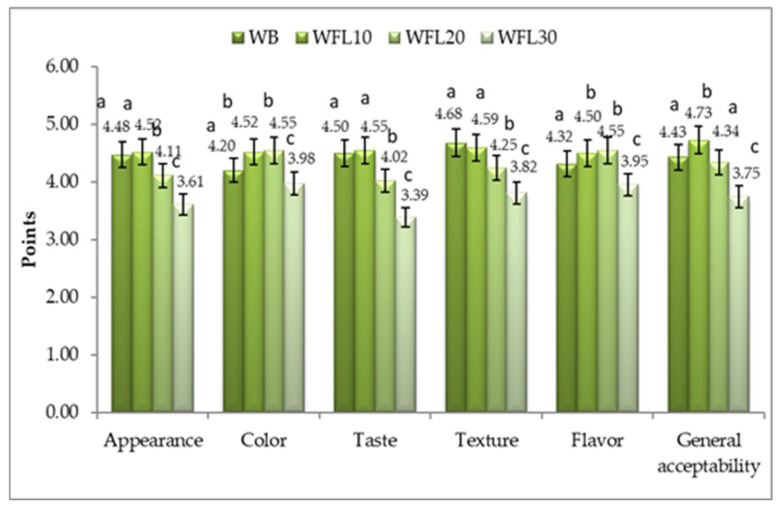
Global values of the sensory evaluation (consumer acceptance) of bread with lupin: WB (control bread); WFL10 (10% lupin flour and 90% wheat flour); WFL20 (20% lupin flour and 80% wheat flour); and WFL30 (30% lupin flour and 70% wheat flour) by using a 5-point hedonic scale (*n* = 44). a,b,c—*t*-test has been used to compare the differences recorded between the samples for each characteristic assessed; data within the same column sharing different superscripts are significantly different (*p* < 0.05).

Evaluation of consumers’ acceptability of lupin bread samples was carried out using sensory evaluation by a panel of 44 assessors (20 male and 24 women) with the help of a five-point hedonic scale. Figure 3 indicates the mean scores for the sensory attributes (appearance, color, texture, taste, flavor, and general acceptability) of the studied lupin bread: WB (control wheat bread); WBL10 (10% lupin flour and 90% wheat flour); WBL20 (20% lupin flour and 80% wheat flour); and WBL30 (30% lupin flour and 70% wheat flour). 

The most highly appreciated was the WBL10 sample with 10% lupin flour, which obtained mean scores of 4.55 for taste and 4.52 for color and 4.73 for general acceptability, (Figure 3), falling within the 4.5–5.00 score range, which indicates high acceptability (HA). In terms of appearance, taste, and general acceptability, the panelists ranked the bread samples in the following order: WBL10 > WB > WBL20 > WBL30 and in terms of flavor and color: WFL20 > WFL10 > WB > WFL30. The WFL20 sample fell within the 3.5–4.49 level of acceptance (acceptable—A) for appearance, taste, texture, and general acceptability and within 4.5–5 (HA) level of acceptance for color and flavor (Figure 3).

The control sample (with wheat flour) was rated with scores similar to the WFL10 sample, scoring on a scale between 4.5 and 5, i.e., highly acceptable (HA), for taste and texture and between 3.5 and 4. 49, i.e., acceptable (A), for appearance, color, flavor, and general acceptability. The lowest rates were obtained by WFL30 sample, with 30% lupin substitution registering values between 2.50 and 3.49 (moderately acceptable) for taste and acceptable (3.5 and 4.49) for all other attributes (Figure 3). The results regarding sensory evaluation of lupin bread are in agreement with the data obtained by Kefale B. and Yetenayet B. (2020) [35].

The addition of different proportions of lupin flour exhibited a substantial effect on the sensory properties of bread samples. The sensory scores for the color of the bread increased once with the incorporation of lupin flour (up to 20% lupin substitution). A light-yellow color given by the natural pigments present in lupin flour was well-appreciated by the evaluators compared to the whitish color of the control sample.

## 4. Conclusions

The global evaluation from the nutritional, phytochemical, sensory, and rheological point of view of the flour composites fortified with lupin and the bread obtained showed that lupin flour can represent a valid vegetal source in baking for a sustainable economy. The nutritional and functional attributes of wheat bread were enhanced using 10–30% lupin flour, but significant shortening of rheological properties, i.e., dough stability, may cause technological issues. The consumers’ acceptability regarding this type of bread indicated that the most highly appreciated was the bread with 10 % lupin flour, but the bread with the addition of 20–30% lupin flour also obtained good scores on certain sensory parameters. 

Our study aims to strengthen the attempt at widening the assorted range of vegetal matrices for use in baking in order to find alternative solutions to cereal flour.

## Figures and Tables

**Table 1:** Recipes for composite lupin bread.

Samples	Ingredients				
	Lupin Flour (g)	Wheat Flour Type 550 (g)	Yeast (g)	Salt (g)	Water * (mL)
WB	-	300	3	4.5	167.4
WBL10	30	270	3	4.5	180
WBL20	60	240	3	4.5	193.5
WBL30	90	210	3	4.5	186.6

* Calculated based on water absorption capacity from Mixolab profile (Supplementary Materials).

**Table 2:** Proximate composition of lupin flour composites and bread.

Sample	Chemical Parameters							
	Moisture (%)	Protein (%)	Increase Compared to the WF (%)	Lipids (%)	Increase Compared to the WF (%)	Ash (%)	Carbohydrates * (g/100 g)	Energy Value ** (kcal/ 100 g)
**Flour Composites**								
LF	6.91 ± 0.05 ^a^	30.95 ± 0.94 ^a^	-	12.42 ± 0.52 ^a^	-	3.67 ± 0.09 ^a^	45.79	418.75
WF	6.76 ± 0.06 ^a^	12.00 ± 0.55 ^b^	-	0.32 ± 0.02 ^b^	-	0.66 ± 0.02 ^b^	80.10	371.30
WFL10	6.80 ± 0.06 ^a^	13.76 ± 0.58 ^c^	14.67	1.66 ± 0.05 ^c^	418.75	1.32 ± 0.05 ^c^	76.30	375.17
WFL20	6.83 ± 0.05 ^a^	16.04 ± 0.66 ^d^	33.67	2.71 ± 0.07 ^d^	746.875	1.00 ± 0.05 ^d^	73.25	381.56
WFL30	6.90 ± 0.06 ^a^	18.35 ± 0.68 ^e^	52.92	4.02 ± 0.09 ^e^	1156.25	1.33 ± 0.06 ^c^	69.23	386.49
**Flour Composites**								
WB	37.92 ± 0.98 ^b^	7.71 ± 0.09 ^a^	-	0.34 ± 0.03 ^a^	-	0.73 ± 0.04 ^a^	52.34	243.23
WBL10	36.34 ± 1.71 ^b^	8.72 ± 0.41 ^b^	13.10	0.92 ± 0.01 ^b^	170.59	0.77 ± 0.02 ^a^	52.21	251.99
WBL20	34.64 ± 1.08 ^b^	10.41 ± 0.47 ^c^	35.02	0.94 ± 0.03 ^b^	176.47	1.15 ± 0.05 ^b^	51.73	257.05
WBL30	32.86 ± 1.130 ^a^	12.24 ± 0.35 ^d^	58.75	0.97 ± 0.05 ^b^	185.29	1.34 ± 0.06 ^c^	51.44	263.44

^a–d^ A *t*-test was used to compare the mean differences registered among samples; data within the same column sharing different superscripts are significantly different (*p* < 0.05); * were calculated according to Equations (1) and (2) using the mean values of equation terms.

**Table 3:** Phytochemical profile of lupin flour composites and bread.

Sample	Phytochemical Parameters					
	Total Polyphenols Content (TPC) (mg GAE/100 g)	Increase Compared to the WF (%)	Total Flavonoids Content (TFC) (mg QE/100 g)	Increase Compared to the WF (%)	Antioxidant Activity (AA) (%)	Increase Compared to the WF (%)
	**Flour Composites**					
LF	128.65 ± 0.66 ^a^	-	12.36 ± 0.11 ^a^	-	93.37 ± 1.53 ^a^	-
WF	53.72 ± 0.38 ^b^	-	4.65 ± 0.07 ^b^	-	52.98 ± 0.24 ^b^	-
WFL10	56.72 ± 0.20 ^c^	5.58	5.37 ± 0.04 ^c^	15.48	70.40 ± 0.42 ^c^	32.88
WFL20	73.03 ± 0.33 ^d^	35.95	5.67 ± 0.02 ^d^	21.94	76.03 ± 0.90 ^d^	43.51
WFL30	85.41 ± 1.94 ^e^	58.99	7.30 ± 0.03 ^e^	56.99	82.37 ± 1.17 ^e^	55.47
	**Composite Bread Variants**					
WB	31.79 ± 0.58 ^a^	-	3.21 ± 0.06 ^a^	-	30.71 ± 3.82 ^a^	-
WBL10	43.19 ± 0.33 ^b^	35.86	4.27 ± 0.03 ^b^	33.02	35.50 ± 0.50 ^a^	15.60
WBL20	60.82 ± 1.37 ^c^	91.32	6.85 ± 0.13 ^c^	113.40	43.64 ± 0.67 ^b^	42.10
WBL30	76.50 ± 0.70 ^d^	140.64	8.54 ± 0.19 ^d^	166.04	54.98 ± 1.21 ^c^	79.03

^a–d^ A *t*-test was used to compare the mean differences registered among samples; data within the same column sharing different superscripts are significantly different (*p* < 0.05).

**Table 4:** Macro and microelements content of lupin flour composites and breads.

Sample	Macro- and Microelements Content (mg/kg)								
	Ca	K	Mg	Fe	Zn	Cu	Cr	Ni	Mn
Flour Composites									
**LF**	290.00 ± 7.07 ^a^	167.67 ± 5.65 ^a^	783.33 ± 14.14 ^a^	22.00 ± 1.41 ^a^	23.35 ± 0.50 ^b^	3.46 ± 0.09 ^a^	0.002 ± 0.0001 ^a^	0.02 ± 0.001 ^a^	136.74 ± 4.99 ^a^
**WF**	225.33 ± 6.60 ^b^	50.23 ± 5.64 ^a^	310.00 ± 6.65 ^b^	5.05 ± 0.01 ^b^	12.79 ± 0.18 ^a^	nd	0.04 ± 0.001 ^b^	nd	2.53 ± 0.001 ^b^
**WFL10**	226.00 ± 6.36 ^b^	70.32 ± 1.41 ^b^	340.67 ± 7.07 ^c^	5.64 ± 0.14 ^c^	21.45 ± 0.62 ^b^	2.31 ± 0.07 ^b^	0.03 ± 0.001 ^b^	0.002 ± 0.0001 ^b^	52.37 ± 2.55 ^c^
**WFL20**	244.63 ± 5.64 ^b^	110.67 ± 2.83 ^c^	481.00 ± 12.16 ^d^	11.58 ± 0.58 ^d^	15.41 ± 0.44 ^c^	2.77 ± 0.147 ^b^	0.01 ± 0.0002 ^c^	0.003 ± 0.0001 ^b^	94.36 ± 3.72 ^d^
**WFL30**	287.66 ± 6.47 ^a^	165.33 ± 5.66 ^a^	615.80 ± 12.73 ^e^	18.23 ± 0.575 ^a^	13.125 ± 0.42 ^a^	3.21 ± 0.21 ^a^	0.003 ± 0.0001 ^a^	0.004 ± 0.0002 ^b^	103.67 ± 4.02 ^e^
**Composite Breads**									
**WB**	129.00 ± 4.24 ^a^	162.33 ± 4.24 ^a^	124.67 ± 5.66 ^a^	11.48 ± 0.58 ^a^	5.92 ± 0.26 ^a^	0.60 ± 0.01 ^a^	0.002 ± 0.0001 ^a^	0.001 ± 0.0001 ^a^	2.95 ± 0.15 ^a^
**WBL10**	262.00 ± 5.66 ^b^	166.67 ± 3.82 ^b^	195.00 ± 6.79 ^b^	11.96 ± 0.42 ^a^	8.42 ± 0.30 ^b^	0.64 ± 0.01 ^a^	0.004 ± 0.0002 ^a,c^	0.004 ± 0.0002 ^b^	46.76 ± 2.93 ^b^
**WBL20**	468.33 ± 11.31 ^c^	167.00 ± 5.66 ^b^	252.00 ± 6.08 ^c^	13.34 ± 0.043 ^b^	10.01 ± 0.34 ^c^	1.56 ± 0.06 ^b^	0.01 ± 0.0003 ^b,c^	0.01 ± 0.0004 ^c^	92.21 ± 4.03 ^c^
**WBL30**	533.00 ± 10.61 ^d^	167.67 ± 5.61 ^b^	403.00 ± 10.32 ^d^	14.88 ± 0.47 ^b^	14.15 ± 0.30 ^d^	1.67 ± 0.06 ^b^	0.01 ± 0.0004 ^d^	0.02 ± 0.001 ^d^	117.37 ± 6.55 ^d^

^a–e^ A *t*-test was used to compare the mean differences registered among samples; data within the same column sharing different superscripts are significantly different (*p* < 0.05).

**Table 5:** Saturated fatty acid (SFA) composition of sample expressed as % of total fatty acids.

Sample	Saturated Fatty Acid Content (%)										
	C4:0	C6:0	C8:0	C10:0	C12:0	C14:0	C16:0	C18:0	C20.0	C22:0	C24:0
Flour Composites											
**LF**	0.15 ± 0.01 ^a^	0.24 ± 0.01 ^a^	0.31 ± 0.01 ^a^	0.24 ± 0.01 ^a^	0.30 ± 0.01 ^a^	1.23 ± 0.03 ^a^	21.38 ± 1.21 ^a^	5.50 ± 0.21 ^a^	1.67 ± 0.04 ^a^	1.40 ± 0.03 ^a^	0.53 ± 0.02 ^a^
**WF**	0.68 ± 0.03 ^b^	0.72 ± 0.04 ^b^	0.21 ± 0.01 ^b^	0.08 ± 0.002 ^b^	0.24 ± 0.01 ^b^	1.12 ± 0.02 ^b^	22.44 ± 1.25 ^b^	12.20 ± 0.65 ^b^	0.51 ± 0.02 ^b^	0.15 ± 0.01 ^b^	0.33 ± 0.01 ^b^
**WFL10**	0.64 ± 0.03 ^b^	0.56 ± 0.03 ^c^	0.23 ± 0.01 ^b^	0.10 ± 0.005 ^b^	0.25 ± 0.01 ^b^	1.15 ± 0.02 ^b,c^	21.93 ± 1.20 ^b^	12.07 ± 0.62 ^b^	0.71 ± 0.03 ^c^	1.07 ± 0.02 ^c^	0.37 ± 0.02 ^b^
**WFL20**	0.41 ± 0.02 ^c^	0.41 ± 0.02 ^d^	0.26 ± 0.02 ^c^	0.13 ± 0.005 ^c^	0.26 ± 0.01 ^b^	1.17 ± 0.02 ^c^	21.71 ± 1.17 ^a,b^	11.44 ± 0.58 ^c^	0.79 ± 0.04 ^c^	1.14 ± 0.03 ^c^	0.38 ± 0.02 ^b^
**WFL30**	0.25 ± 0.01 ^d^	0.35 ± 0.02 ^e^	0.28 ± 0.02 ^c^	0.18 ± 0.01 ^d^	0.28 ± 0.02 ^a,b^	1.21 ± 0.02 ^a^	21.52 ± 1.15 ^a^	10.36 ± 0.55 ^c^	0.91 ± 0.04 ^d^	1.24 ± 0.04 ^d^	0.43 ± 0.02 ^c^
**Composite Breads**											
**WB**	0.36 ± 0.02 ^a^	0.30 ± 0.02 ^a^	0.17 ± 0.01 ^a^	0.05 ± 0.001 ^a^	0.27 ± 0.01 ^a^	1.14 ± 0.04 ^a^	22.52 ± 1.25 ^a^	15.01 ± 0.80 ^a^	0.38 ± 0.02 ^a^	0.11 ± 0.005 ^a^	0.18 ± 0.01 ^a^
**WBL10**	0.35 ± 0.01 ^a^	0.22 ± 0.01 ^b^	0.19 ± 0.01 ^a,b^	0.08 ± 0.002 ^a^	0.27 ± 0.01 ^a^	1.15 ± 0.05 ^a^	22.14 ± 1.22 ^a^	11.85 ± 0.75 ^b^	0.55 ± 0.03 ^b^	0.16 ± 0.01 ^b^	0.31 ± 0.02 ^b^
**WBL20**	0.32 ± 0.01 ^a^	0.20 ± 0.01 ^b^	0.20 ± 0.01 ^a,b^	0.15 ± 0.005 ^b^	0.23 ± 0.01 ^a^	1.18 ± 0.05 ^a^	21.82 ± 1.07 ^b^	10.60 ± 0.60 ^b^	1.10 ± 0.05 ^c^	1.07 ± 0.01 ^a^	0.33 ± 0.02 ^b^
**WBL30**	0.25 ± 0.01 ^b^	0.14 ± 0.01 ^c^	0.22 ± 0.01 ^b^	0.23 ± 0.01 ^c^	0.19 ± 0.01 ^b^	1.21 ± 0.06 ^b^	21.75 ± 1.06 ^b^	9.12 ± 0.55 ^c^	1.41 ± 0.06 ^d^	1.15 ± 0.01 ^b^	0.49 ± 0.03 ^c^

^a–d^ A *t*-test was used to compare the mean differences registered among samples; data within the same column sharing different superscripts are significantly different (*p* < 0.05) flour composites.

**Table 6:** Unsaturated fatty acid (UFA) composition of sample expressed as % of total fatty acids.

Sample	Unsaturated Fatty Acid Content (%)										
	C16:1n9	C16:1n7	C18:1n11	C18:1n9	C18:1n7	C18:2n6	C18:3n3	C20:1n9	C20:1n7	C18.2n7	C22:1n9
Flour Composites											
**LF**	0.13 ± 0.01 ^a^	0.49 ± 0.02 ^a^	0.74 ± 0.04 ^a^	40.58 ± 2.54 ^a^	2.16 ± 0.12 ^a^	18.64 ± 1.44 ^a^	0.03 ± 0.001 ^a^	3.52 ± 0.22 ^a^	0.16 ± 0.01 ^a^	0.26 ± 0.02 ^a^	0.25 ± 0.01 ^a^
**WF**	0.67 ± 0.03 ^b^	nd	nd	17.14 ± 1.34 ^b^	nd	40.92 ± 2.47 ^b^	1.58 ± 0.07 b	1.63 ± 0.08 ^b^	nd	nd	nd
**WFL10**	0.61 ± 0.03 ^b^	0.05 ± 0.002 ^b^	0.10 ± 0.005 ^b^	20.81 ± 1.58 ^c^	0.05 ± 0.002 b	36.49 ± 2.05 ^c^	1.26 ± 0.06 ^c^	1.77 ± 0.07 ^b,c^	0.01 ± 0.001 ^b^	0.03 ± 0.001 ^b^	0.04 ± 0.002 ^b^
**WFL20**	0.52 ± 0.02 ^c^	0.11 ± 0.005 ^c^	0.36 ± 0.01 ^c^	25.05 ± 1.98 ^d^	0.22 ± 0.01 ^c^	32.49 ± 1.68 ^c,d^	1.08 ± 0.05 ^d^	1.89 ± 0.09 ^c,d^	0.05 ± 0.002 ^c^	0.07 ± 0.002 ^b,c^	0.10 ± 0.005 ^c^
**WFL30**	0.44 ± 0.02 ^d^	0.19 ± 0.01 ^d^	0.48 ± 0.02 ^d^	28.78 ± 2.05 ^e^	0.53 ± 0.02 ^d^	28.55 ± 1.56 ^d^	0.75 ± 0.03 ^e^	2.03 ± 0.09 ^d^	0.09 ± 0.004 ^d^	0.10 ± 0.005 ^c^	0.14 ± 0.01 ^d^
**Composite Breads**											
**WB**	0.55 ± 0.03 ^a^	nd	nd	16.83 ± 0.91 ^a^	nd	39.60 ± 2.22 ^a^	1.19 ± 0.04 ^a^	1.19 ± 0.04 ^a^	nd	nd	nd
**WBL10**	0.52 ± 0.02 ^a^	0.03 ± 0.001 ^a^	0.06 ± 0.003 ^a^	21.44 ± 1.15 ^b^	0.09 ± 0.01 ^a^	38.26 ± 2.06 a	1.02 ± 0.04 ^b^	1.33 ± 0.05 ^b^	0.04 ± 0002 a	0.07 ± 0.003 ^a^	0.06 ± 0.002 ^a^
**WBL20**	0.40 ± 0.02 ^b^	0.07 ± 0.002 ^b^	0.32 ± 0.01 ^b^	25.76 ± 1.54 ^c^	0.12 ± 0.01 ^b^	33.04 ± 1.86 ^b^	0.98 ± 0.03 ^b^	1.58 ± 0.07 c	0.09 ± 0.004 ^b^	0.08 ± 0.004 ^a^	0.10 ± 0.005 ^b^
**WBL30**	0.36 ± 0.01 ^c^	0.20 ± 0.01 ^c^	0.45 ± 0.02 ^c^	29.93 ± 1.62 ^d^	0.15 ± 0.01 ^c^	29.46 ± 1.64 ^c^	0.51 ± 0.02 ^c^	2.03 ±0.10 ^d^	0.10 ± 0.005 ^b^	0.13 ± 0.005 ^b^	0.11 ± 0.005 ^b^

^a–d^ A *t*-test was used to compare the mean differences registered among samples; data within the same column sharing different superscripts are significantly different (*p* < 0.05) flour composites.

**Table 7:** Fatty acid classes (% of total fatty acids) fatty acid ratio and quality indices, IA and IT.

	SFAs	MUFAs	PUFAs	UFAs	UFA/SFA	PUFA/SFA	AI	TI
Flour Composites								
**LF**	32.95	48.03	18.93	66.96	2.03	0.57	0.40	0.84
**WF**	38.68	19.44	42.50	61.94	1.60	1.10	0.44	1.02
**WFL10**	39.08	23.44	37.78	61.22	1.57	0.97	0.44	1.04
**WFL20**	38.10	28.30	33.64	61.94	1.63	0.88	0.43	1.02
**WFL30**	37.01	32.68	29.40	62.08	1.68	0.79	0.43	1.01
**Composite Breads**								
**WB**	40.49	18.57	40.79	59.36	1.47	1.01	0.46	1.18
**WBL10**	37.07	23.57	39.35	62.92	1.70	1.06	0.43	1.03
**WBL20**	37.16	28.44	34.10	62.54	1.68	0.92	0.43	1.00
**WBL30**	36.20	33.33	30.10	63.43	1.75	0.83	0.42	0.97

**Table 8:** Bread quality indicators for control wheat bread (WB); WBL10 (10% lupin flour and 90% wheat flour); WBL20 (20% lupin flour and 80% wheat flour); and WBL30 (30% lupin flour and 70% wheat flour).

Indicator	M.U	WB	WBL10	WBL20	WBL30
Volume	cm^3^/100 g	434 ± 1.73 ^a^	431 ± 1 ^a,c^	428 ± 1.73 ^b,c^	402 ± 1 ^d^
Decrease compared to the WB (%)			−0.69	−1.38	−7.37
Porosity	%	65.62 ± 1.35 ^a^	61.08 ± 1.32 ^b^	53.48 ± 1.18 ^a^	42.48 ± 1.08 ^b^
Decrease compared to the WB (%)			−6.92	−18.50	−35.26
Elasticity	%	71 ± 2 ^a^	70 ± 2 ^a,c^	68 ± 1.73 ^a,d^	63 ± 2 ^b^
Decrease compared to the WB (%)			−1.41	−4.23	−11.27
Ratio between high and diameter (H/D)	-	0.63 ± 0.002 ^a^	0.61 ± 0.001 ^b^	0.56 ± 0.002 ^c^	0.51 ± 0.002 ^d^
Decrease compared to the WB (%)			−3.17	−11.11	−19.05

^a–d^ A *t*-test was used to compare the mean differences registered among samples; data within the same column sharing different superscripts are significantly different (*p* < 0.05).

**Table 9:** The primary parameters of the lupin flour composites.

Samples	WA (%)	ST (min)	C1	C2	C3	C4	C5	α (nm/min)	β (nm/min)	γ (nm/min)
WF	55.8	9.52	1.19	0.54	2.037	1.86	3.29	−0.07	0.31	−0.05
WFL10	60.0	6.62	1.12	0.46	1.74	1.69	2.82	−0.06	0.30	−0.002
WFL20	64.5	5.68	1.12	0.41	1.56	1.52	2.49	−0.06	0.43	−0.02
WFL30	62.2	5.47	1.18	0.41	1.27	1.42	2.30	−0.07	0.19	−0.03

**Table 10:** Profiler Mixolab indices expressed on a scale from 0–9 (Mixolab Index).

Samples	Absorption Index (WAI)	Mixing Index (MI)	Gluten+ Index (GI)	Viscosity Index (VI)	Amylolysis Index (AI)	Retrogradation Index (RI)
WF	2	6	4	7	8	8
WFL10	7	2	5	4	7	7
WFL20	8	2	4	2	6	7
WFL30	8	2	3	1	6	6

## Data Availability

The report of the analyzes performed for the samples in the paper can be found at the Interdisciplinary Research Platform (PCI) belonging to the Banat University of Agricultural Sciences and Veterinary Medicine “King Michael I of Romania” from Timisoara.

## Supplementary Materials

The following supporting information can be downloaded at: https://www.mdpi.com/article/10.3390/foods11142067/s1, Supplementary File.

## References

[1] Starkute V., Bartkiene E., Bartkevics V., Rusko J., Zadeike D., Juodeikiene G. (2016). Amino acids profile and antioxidant activity of different *Lupinus angustifolius* seeds after solid state and submerged fermentations. J. Food Sci. Technol..

[2] Ayyash M., Johnson S.K., Liu S.Q., Mesmari N., Dahmani S., Al Dhaheri A.S., Kizhakkayil J. (2019). In vitro investigation of bioactivities of solid-state 485 fermented lupin, quinoa and wheat using *Lactobacillus* spp.. Food Chem..

[3] https://www.tridge.com/intelligences/lupin-bean/production.

[4] https://worldpopulationreview.com/country-rankings/wheat-production-by-country.

[5] Gulisano A., Alves S., Martins J.N., Trindade L.M. (2019). Genetics and Breeding of *Lupinus mutabilis*: An Emerging Protein Crop. Front. Plant Sci..

[6] Duranti M., Consonni A., Magni C., Sessa F., Scarafoni A. (2008). The major proteins of lupin seed: Characterisation and molecular properties for use as functional and nutraceutical ingredients. Trends Food Sci. Technol..

[7] Alomari D.Z., Abdul-Hussain S. (2013). Effect of Lupin Flour Supplementation on Chemical, Physical and Sensory Properties of Mediterranean Flat Bread. Int. J. Food Sci. Nutr. Eng..

[8] Chiofalo B., Lo Presti V., Chiofalo V., Gresta F. (2012). The productive traits, fatty acid profile and nutritional indices of three lupin (*Lupinus* spp.) species cultivated in a Mediterranean environment for the livestock. Anim. Feed. Sci. Technol..

[9] Khan M.K., Karnpanit W., Nasar-Abbas S.M., Huma Z.-E., Jayasena V. (2015). Phytochemical composition and bioactivities of lupin: A review. Int. J. Food Sci. Technol..

[10] Jayasena V., Nasar-Abbas S.M. (2012). Development and quality evaluation of high-protein and high-dietary-fiber pasta using lupin flour. J. Texture Stud..

[11] Wandersleben T., Morales E., Burgos-Díaz C., Barahona T., Labra E., Rubilar M., Salvo-Garrido H. (2018). Enhancement of functional and nutritional properties of bread using a mix of natural ingredients from novel varieties of flaxseed and lupin. LWT—Food Sci. Technol..

[12] Boukid F., Zannini E., Carini E., Vittadini E. (2019). Pulses for bread fortification: A necessity or a choice?. Trends Food Sci. Technol..

[13] Schlegel K., Lidzba N., Ueberham E., Eisner P., Schweiggert-Weisz U. (2021). Fermentation of Lupin Protein Hydrolysates—Effects on Their Functional Properties, Sensory Profile and the Allergenic Potential of the Major Lupin Allergen Lup an 1. Foods.

[14] Doxastakis G., Zafiriadis I., Irakli M., Marlani H., Tananaki C. (2002). Lupin, soya and triticale addition to wheat flour doughs and their effect on rheological properties. Food Chem..

[15] Hrušková M., Svec I., Kubalová M., Bachanová M. Lupin Addition Effect on Wheat Flour, Dough and Bread Properties. Proceedings of the CIGR Section VI International Symposium on Food Processing, Monitoring Technology in Bioprocesses and Food Quality Management.

[16] Rusu I.E., Marc R.A., Muresan C.C., Muresan A.E., Muresan V., Pop C.R., Chis M.S., Man S.M., Filip M.R., Onica B.-M. (2021). Hemp (*Cannabis sativa* L.) Flour-Based Wheat Bread as Fortified Bakery Product. Plants.

[17] Pop A., Păucean A., Socaci S.A., Alexa E., Man S.M. (2020). Quality characteristics and volatile profile of macarons modified with walnut oilcake by-product. Molecules.

[18] Chiş M.S., Pop A., Păucean A., Socaci S.A., Alexa E., Man S.M., Bota M. (2020). Fatty acids, volatile and sensory profile of multigrain biscuits enriched with spent malt rootles. Molecules.

[19] Vasilica B.B., Chis M.S., Alexa E., Pop C., Paucean A., Man S., Igual M., Haydee K.M., Dalma K.E., Stanila S. (2022). The Impact of Insect Flour on Sourdough Fermentation-Fatty Acids, Amino-Acids, Minerals and Volatile Profile. Insects.

[20] Association of Official Analytical Chemists (AOAC) (2000). Official Methods of Analysis of AOAC International.

[21] Obistioiu D., Cocan I., Tîrziu E., Herman V., Negrea M., Cucerzan A., Neacsu A.-G., Cozma A.L., Nichita I., Hulea A. (2021). Phytochemical Profile and Microbiological Activity of Some Plants Belonging to the Fabaceae Family. Antibiotics.

[22] Lazăr R.N., Alexa E., Obiștioiu D., Cocan I., Pătruică S. (2022). The Effect of the Use of Essential Oils in the Feed of Bee Families on Honey Chemical Composition and Antimicrobial Activity. Appl. Sci..

[23] Ciulca S., Roma G., Alexa E., Radulov I., Cocan I., Madosa E., Ciulca A. (2021). Variation of Polyphenol Content and Antioxidant Activity in Some Bilberry (*Vaccinium myrtillus* L.) Populations from Romania. Agronomy.

[24] Poșta D.S., Radulov I., Cocan I., Berbecea A.A., Alexa E., Hotea I., Iordănescu O.A., Băla M., Cântar I.C., Rózsa S. (2022). Hazelnuts (*Corylus avellana* L.) from Spontaneous Flora of the West Part of Romania: A Source of Nutrients for Locals. Agronomy.

[25] Alexa E. (2008). Vegetal Food Technology.

[26] (2007). Romanian Standard for Bread, confectionery andbakery specialties—Methods of Analysis.

[27] Mixolab Applications Handbook (2009). Rheological and Enzymatic Analysis.

[28] Bánfalvi Á., Németh R., Bagdi A., Gergely S., Rakszegi M., Bedő Z., Láng L., Vidab G., Tömösközia S. (2020). A novel approach to the characterization of old wheat *(Triticum aestivum* L.) varieties by complex rheological analysis. J. Sci. Food Agric..

[29] Bojnanská T., Musilová J., Vollmannová A. (2021). Effects of Adding Legume Flours on the Rheological and Breadmaking Properties of Dough. Foods.

[30] Pertuzatti P.B., Esteves S.M.R., Alves J.E., Lima L.C., Borges J.E. (2015). Sensory Evaluation of Bakery and Confectionery Products Prepared through Semi-Industrial and Artisanal Processes. Am. J. Food Sci. Technol..

[31] Lqari H., Vioque J., Pedroche J., Millán F. (2002). *Lupinus angustifolius* protein isolates: Chemical composition, functional properties and protein characterization. Food Chem..

[32] Jiménez Martínez C., Hernández Sánchez H., Dávila Ortiz G. (2003). Lupins: An Alternative for Debittering and Utilization in Foods. Food Science and Food Biotechnology.

[33] El-Adawy T.A., Rahma E.H., El-Bedawey A.A., Gafar A.F. (2001). Nutritional potential and functional properties of sweet and bitter lupin seed protein isolates. Food Chem..

[34] Muranyi I.S., Volke D., Hoffmann R., Eisner P., Herfellner T., Brunnbauer M., Koehler P., Schweiggert-Weisz U. (2016). Protein distribution in lupin protein isolates from *Lupinus angustifolius* L. prepared by various isolation techniques. Food Chem..

[35] Kefale B., Yetenayet B. (2020). Evaluation of bread prepared from composite flour of sweet lupin and Bread wheat variety. Acad. Res. J. Agric. Sci. Res..

[36] Hall R.S., Johnson S.K., Baxter A.L., Ball M.J. (2005). Lupin kernel fibre-enriched foods beneficially modify serum lipids in men. Eur. J. Clin. Nutr..

[37] Păucean A. (2017). Modern Trends in Increasing the Nutritional Value of Wheat Flour and Bakery Products.

[38] Serventi L., Vittadini E., Vodovotz Y. (2018). Effect of chickpea protein concentrate on the loaf quality of composite soy-wheat bread. LWT.

[39] Previtali M.A., Mastromatteo M., De Vita P., Ficco D.B.M., Conte A., Del Nobile M.A. (2014). Effect of the lentil flour and hydrocolloids on baking characteristics of wholemeal durum wheat bread. Int. J. Food Sci. Technol..

[40] Dalaram I.S. (2017). Evaluation of total polyphenol content and antioxidant capacity of different verity lupin seeds. Potravin. Slovak J. Food Sci..

[41] Liezhou Z., Zhongxiang F., Wahlqvist M.L., Hodgson J.M., Johnson S.K. (2021). Multi-response surface optimisation of extrusion cooking to increase soluble dietary fibre and polyphenols in lupin seed coat. LWT.

[42] Karamać M., Orak H.H., Amarowicz R., Orak A., Piekoszewski W. (2018). Phenolic contents and antioxidant capacities of wild and cultivated white lupin (*Lupinus albus* L.) seeds. Food Chem..

[43] Vollmannova A., Lidikova J., Musilova J., Snirc M., Bojnanska T., Urminska D., Tirdilova I., Zetochova E. (2021). White Lupin as a Promising Source of Antioxidant Phenolics for Functional Food Production. J. Food Qual..

[44] Ghazal G., Shahhat I., Shahhat A. (2014). Polyphenols, Flavonoids, Carotenoids and Antioxidant activity of lupin (*Lupinus termis* L.) seeds affected by vitamin C, vitamin B 3 and turmeric Rhizomes Extract. Adv. Environ. Biol..

[45] Li Y., Ma D., Sun D., Chenyang W., Zhang J., Xie Y., Guo T. (2015). Total phenolic, flavonoid content, and antioxidant activity of flour, noodles, and steamed bread made from different colored wheat grains by three milling methods. Crop J..

[46] Yaver E., Bilgiçli N. (2021). Development of quality characteristics of pasta enriched with lupin (*Lupinus albus* L.) flour and resistant starch type 4. Tekirdağ Ziraat Fakültesi Derg..

[47] Aslan M., Bilgiçli N. (2021). Improvement of functional cake formulation with fermented soy (*Glycine max*) and lupin (*Lupinus albus* L.) powders. Int. J. Gastron. Food Sci..

[48] Buszewski B., Rafińska K., Cvetanović A., Walczak J., Krakowska A., Joanna R., Zeković Z. (2019). Phytochemical analysis and biological activity of *Lupinus luteus* seeds extracts obtained by supercritical fluid extraction. Phytochem. Lett..

[49] Brandolini A., Glorio P.P., Estivi L., Locatelli N., Córdova-Ramos J., Hidalgo A. (2021). Tocopherols, carotenoids and phenolics changes during Andean lupin (*Lupinus mutabilis* Sweet) seeds processing. J. Food Compos. Anal..

[50] Yaver E., Bilgiçli N. (2021). Ultrasound-treated lupin (*Lupinus albus* L.) flour: Protein- and fiber-rich ingredient to improve physical and textural quality of bread with a reduced glycemic index. LWT.

[51] Yaver E., Bilgiçli N. (2021). Effect of ultrasonicated lupin flour and resistant starch (type 4) on the physical and chemical properties of pasta. Food Chem..

[52] Uauy R., Gattas V., Yáñez E. (1995). Sweet lupins in human nutrition. World Rev. Nutr. Diet..

[53] Andrzejewska J., Ignaczak S., Barzyk P. (2016). Oil content and fatty acid profile in seeds of Polish breeding lines and cultivars of legumes. Acta Sci. Pol. Agric..

[54] Alamri M.S. (2012). Characterization of lupin seed oils extracted from bitter and sweet types. Pak. J. Food Sci..

[55] Zengin G., Nithiyanantham S., Sarikurkcu C., Suysal E., Ceylan R., Ramya K.S., Maskovic P., Aktumsek A. (2017). Identification of phenolic profiles, fatty acid compositions, antioxidant activities, and enzyme inhibition effects of seven wheat cultivars grown in Turkey: A phytochemical approach for their nutritional value. Int. J. Food Prop..

[56] Nikolic N., Radulovic N., Momcilovic B., Nikolic G., Lazic M., Todorovic Z. (2008). Fatty acids composition and rheology properties of wheat and wheat and white or brown rice Xour mixture. Eur. Food Res. Technol..

[57] Burr M.L., Fehily A.M., Gilbert J.F., Rogers S., Holliday R.M., Sweetnam P.M., Elwood P.C., Deadman N.M. (1989). Effects of changes in fat, fish, and fibre intakes on death and myocardial reinfarction: Diet and reinfarction trial (DART). Lancet.

[58] Fehily A.M. (1993). Essential Fatty Acids and Vascular Disease. Vasc. Med. Rev..

[59] Ulbricht T., Southgate D. (1991). Coronary heart disease: Seven dietary factors. Lancet.

[60] Chavan J.K., Kadam S.S. (1993). Nutritional enrichment of barley products by supplementation with non wheat flours. CRC Critical. Rev. Food Sci. Nutr..

[61] Codină G.G., Marineac A.R., Todosi-Sănduleac E. (2016). The influence of lupin flour addition on bread quality. Food Environ. Saf..

[62] Antanas S., Lazureanu A., Alexa E., Negrea M. (2013). Researches regarding the impact of agro-technique measures upon amino acids content in different triticosecale varieties. J. Hortic. For. Biotechnol..

