# Supplementary material for: Lupin (Lupinus spp.)-Fortified Bread: A Sustainable, Nutritionally, Functionally, and Technologically Valuable Solution for Bakery
Source: Foods. 2022 Jul 12;11(14):2067. doi: 10.3390/foods11142067 (PMC9316204; doi:10.3390/foods11142067)
Supplement: Supplementary file 1 [file foods-11-02067-s001.zip › foods-1795633-supplementary.pdf]

Mixolab

CHOPIN Technologies
20 AV. MARCELLIN BERTHELOT
Z.I. DU VAL DE SEINE
92390 VILLENEUVE LA GARENNE
FRANCE

Tests - 115C_9

Date : 25/01/2022 **Hour :** 13:03

Sample :

Water absorption 60.0 % base 14% (b14)

Moisture content 9.90 %

Index: 7-25-477

Protocol : Chopin+

Dough weight : 75.0 g

Water tank temperature : 30.0 °C

Mixing speed : 80 rpm

α :	-0.062	Nm/min
β :	0.304	Nm/min
γ :	-0.002	Nm/min

	Time (min)	Torque (Nm)	Temp. Dough(°C)	Amplitude (Nm)	Stability (min)
C1	4.67	1.124	30.9	0.066	6.62
CS	8.00	0.948	31.3		8.77
C2	17.55	0.460	57.6		
C3	27.07	1.737	84.5		
C4	30.30	1.691	87.4		
C5	45.02	2.816	56.6		

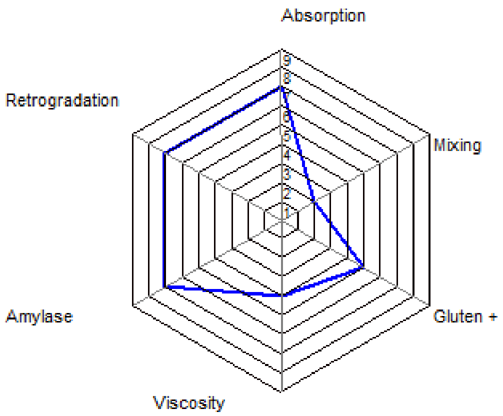

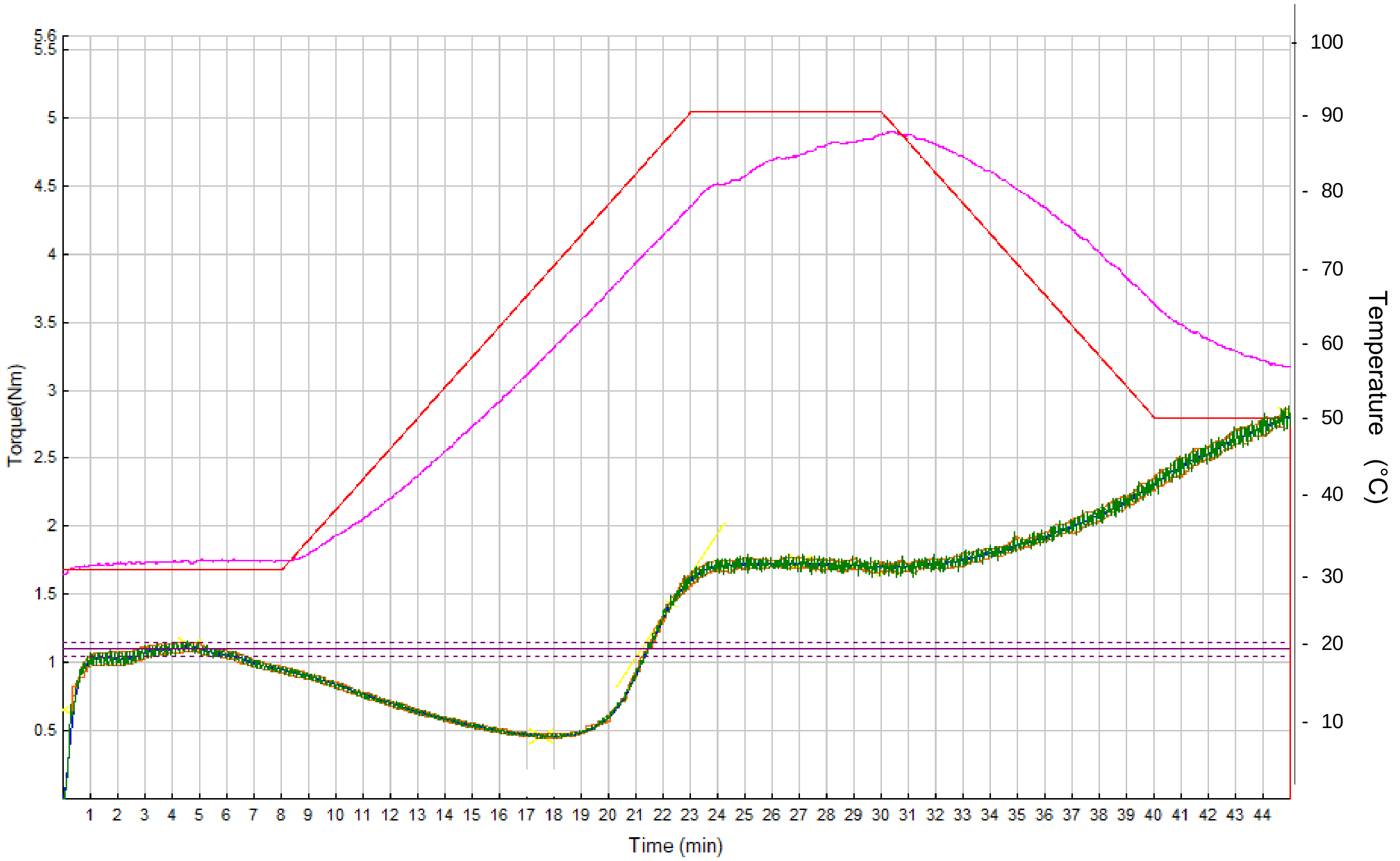

Mixolab

CHOPIN Technologies
20 AV. MARCELLIN BERTHELOT
Z.I. DU VAL DE SEINE
92390 VILLENEUVE LA GARENNE
FRANCE

Tests - 115C_9

Laboratory temperature		°C	Mill			
Lab hygrometry		%	Extraction rate		%	
Moisture content	9.9	%	P		mmH2O	
Proteins		%	L		mm	
Damaged starch		UCD	G			
Zeleny		ml	W		10E-4J	
Ash		%	P/L			
Gluten		%	Ie		%	
Falling number		s	Water absorption	60.0 %		Base 14% (b14)

Mixolab

CHOPIN Technologies
20 AV. MARCELLIN BERTHELOT
Z.I. DU VAL DE SEINE
92390 VILLENEUVE LA GARENNE
FRANCE

Tests - 115C_10

Date : 25/01/2022 **Hour :** 11:42

Sample :

Water absorption 64.5 % base 14% (b14)

Moisture content 11.50 %

Index: 8-24-267

Protocol : Chopin+

Dough weight : 75.0 g

Water tank temperature : 30.0 °C

Mixing speed : 80 rpm

α :	-0.062	Nm/min
β :	0.430	Nm/min
γ :	-0.022	Nm/min

	Time (min)	Torque (Nm)	Temp. Dough(°C)	Amplitude (Nm)	Stability (min)
C1	4.00	1.122	30.8	0.067	5.68
CS	8.00	0.911	31.0		8.33
C2	18.07	0.410	59.8		
C3	25.20	1.560	81.8		
C4	30.13	1.516	87.0		
C5	45.00	2.485	56.2		

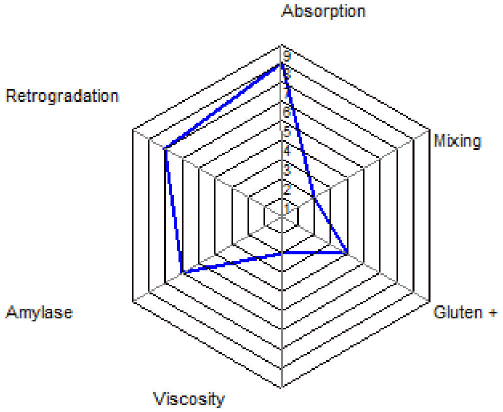

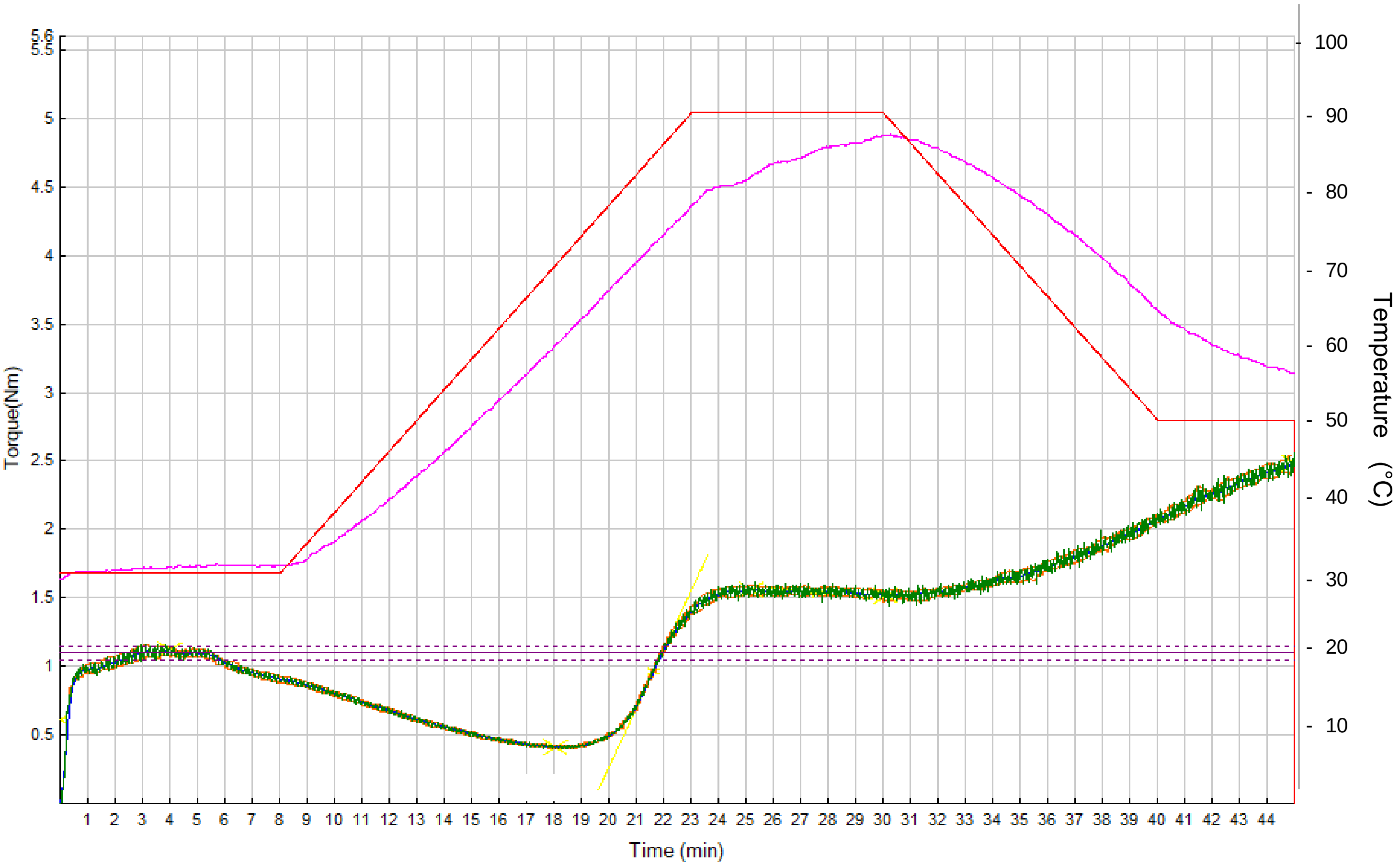

Tests Mixing 1/2 Device N° 166 Version4.0.8+3.50A TNC

Mixolab

CHOPIN Technologies
20 AV. MARCELLIN BERTHELOT
Z.I. DU VAL DE SEINE
92390 VILLENEUVE LA GARENNE
FRANCE

Tests - 115C_10

Laboratory temperature		°C	Mill			
Lab hygrometry		%	Extraction rate			%
Moisture content	11.5	%	P			mmH2O
Proteins		%	L			mm
Damaged starch		UCD	G			
Zeleny		ml	W			10E-4J
Ash		%	P/L			
Gluten		%	Ie			%
Falling number		s	Water absorption	64.5	%	Base 14% (b14)

Mixolab

CHOPIN Technologies
20 AV. MARCELLIN BERTHELOT
Z.I. DU VAL DE SEINE
92390 VILLENEUVE LA GARENNE
FRANCE

Tests - 115_11

Date : 25/01/2022 **Hour :** 14:30

Sample :

Water absorption 62.2 % base 14% (b14)

Moisture content 9.20 %

Index: 8-23-166

Protocol : Chopin+

Dough weight : 75.0 g

Water tank temperature : 30.0 °C

Mixing speed : 80 rpm

α :	-0.070	Nm/min
β :	0.194	Nm/min
γ :	-0.030	Nm/min

	Time (min)	Torque (Nm)	Temp. Dough(°C)	Amplitude (Nm)	Stability (min)
C1	4.90	1.184	31.2	0.055	5.47
CS	8.00	0.945	31.2		8.1
C2	18.60	0.408	61.4		
C3	23.00	1.268	77.5		
C4	30.00	1.422	86.7		
C5	45.02	2.298	56.3		

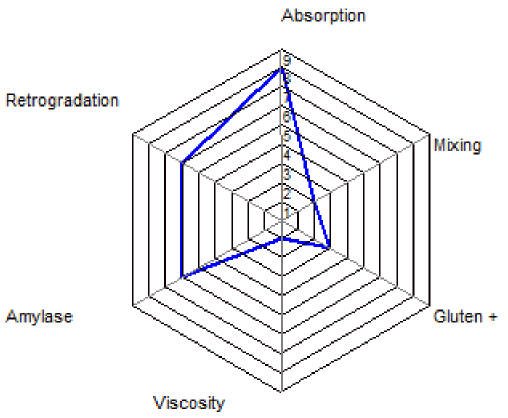

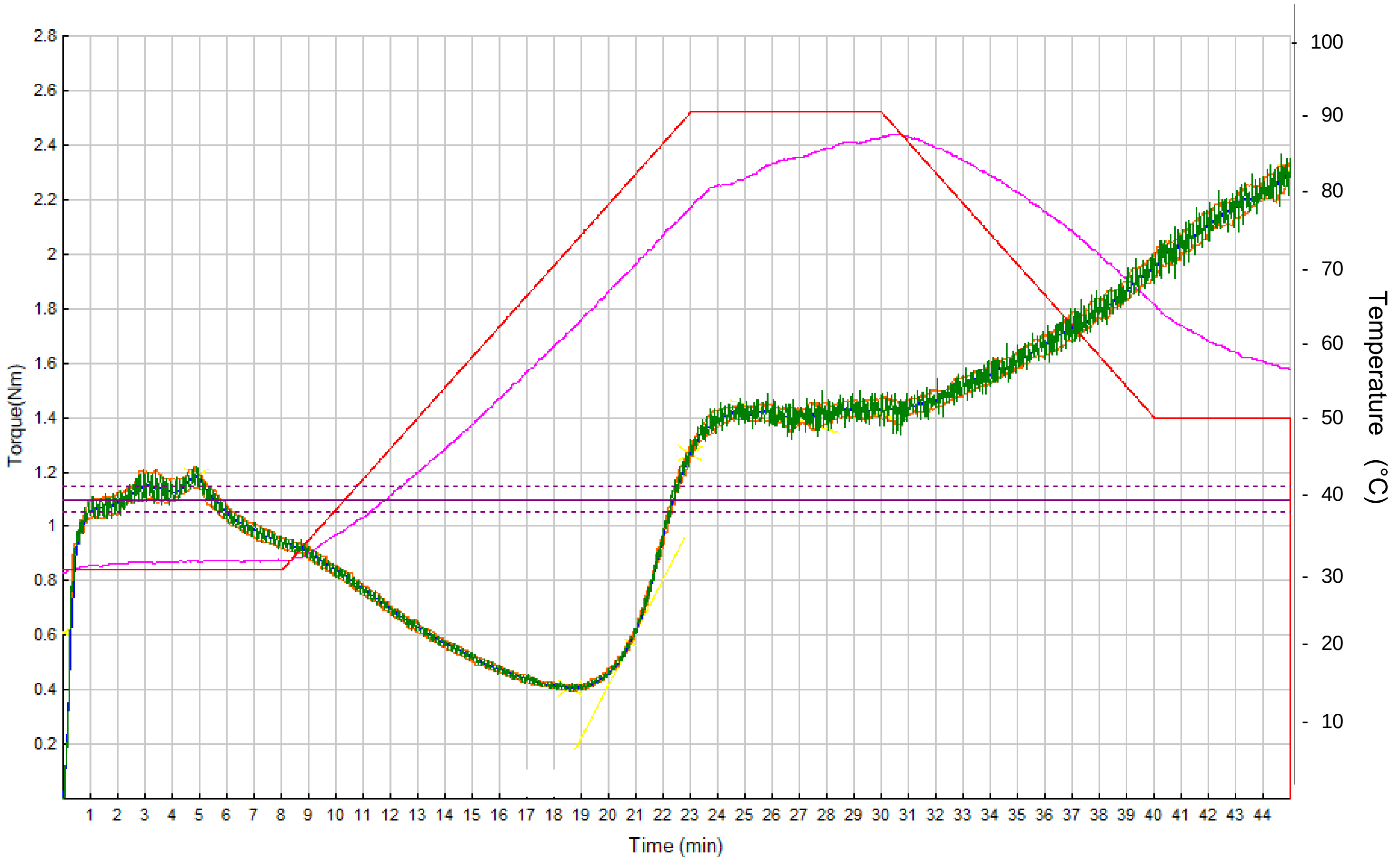

Mixolab

CHOPIN Technologies
20 AV. MARCELLIN BERTHELOT
Z.I. DU VAL DE SEINE
92390 VILLENEUVE LA GARENNE
FRANCE

Tests - 115_11

Laboratory temperature		°C	Mill		
Lab hygrometry		%	Extraction rate		%
Moisture content	9.2	%	P		mmH2O
Proteins		%	L		mm
Damaged starch		UCD	G		
Zeleny		ml	W		10E-4J
Ash		%	P/L		
Gluten		%	Ie		%
Falling number		s	Water absorption	62.2 %	Base 14% (b14)
